# Variation in sexual dimorphism in a wind‐pollinated plant: the influence of geographical context and life‐cycle dynamics

**DOI:** 10.1111/nph.16050

**Published:** 2019-08-12

**Authors:** Gemma Puixeu, Melinda Pickup, David L. Field, Spencer C. H. Barrett

**Affiliations:** ^1^ Institute of Science and Technology Austria Am Campus 1 Klosterneuburg 3400 Austria; ^2^ Department of Ecology and Evolutionary Biology University of Toronto 25 Willcocks St. Toronto ON M5S 3B2 Canada; ^3^ School of Science Edith Cowan University 270 Joondalup Drive Joondalup WA 6027 Australia

**Keywords:** dioecy, geographical variation, *Rumex hastatulus*, sex‐specific selection, sexual dimorphism, wind pollination

## Abstract

- Understanding the mechanisms causing phenotypic differences between females and males has long fascinated evolutionary biologists. An extensive literature exists on animal sexual dimorphism but less information is known about sex differences in plants, particularly the extent of geographical variation in sexual dimorphism and its life‐cycle dynamics.
- Here, we investigated patterns of genetically based sexual dimorphism in vegetative and reproductive traits of a wind‐pollinated dioecious plant, *Rumex hastatulus,* across three life‐cycle stages using open‐pollinated families from 30 populations spanning the geographic range and chromosomal variation (XY and XY_1_Y_2_) of the species.
- The direction and degree of sexual dimorphism was highly variable among populations and life‐cycle stages. Sex‐specific differences in reproductive function explained a significant amount of temporal change in sexual dimorphism. For several traits, geographical variation in sexual dimorphism was associated with bioclimatic parameters, likely due to the differential responses of the sexes to climate. We found no systematic differences in sexual dimorphism between chromosome races.
- Sex‐specific trait differences in dioecious plants largely result from a balance between sexual and natural selection on resource allocation. Our results indicate that abiotic factors associated with geographical context also play a role in modifying sexual dimorphism during the plant life‐cycle.

## Introduction

Trait differences between females and males (sexual dimorphism) reflect sex‐specific optima related to their different reproductive roles (Darwin, 1871; Andersson, 1994). In dioecious plants, the strength and direction of sex‐specific selection can vary within species, providing opportunities to examine the genetic and evolutionary drivers of sexual dimorphism (Lloyd & Webb, 1977; Delph, 1999; Geber *et al*., 1999; Barrett & Hough, 2013). Mechanisms of pollen and seed dispersal may mediate the strength of sex‐specific selection because female and male components interact indirectly through biotic or abiotic vectors (Lloyd & Webb, 1977; Moore & Pannell, 2011). Male‐male competition can be particularly intense in wind‐pollinated species, compared with animal‐pollinated systems, because flowers are commonly uniovulate (Friedman & Barrett, 2011). Consequently, conspicuous sexual dimorphism is predicted to evolve for traits related to pollination success in anemophilous species including plant height, flower number and inflorescence deployment (Eppley & Pannell, 2007; Friedman & Barrett, 2009; Tonnabel *et al*., 2019). Specifically, males are expected to invest in fewer, but larger and taller inflorescences, whereas females are predicted to have flowers spread throughout the air stream and distributed across more inflorescences. Yet sexual selection may also interact with the different resource requirements of the sexes to influence the level and direction of sexual dimorphism. For example, whereas males may have higher nitrogen demands for pollen production, females require a greater investment in photosynthetic tissues to produce carbon for seeds and fruits (Delph, 1999; Harris & Pannell, 2008), which may result in females having higher vegetative investment (Teitel *et al*., 2016). Consequently, sex‐specific trait differences in anemophilous plants may reflect both wind‐mediated selection for proficient pollen and seed dispersal, and optimal resource allocation between vegetative and reproductive structures.

Variation in sexual dimorphism within species may occur at both temporal and geographical scales (Lloyd & Webb, 1977; Barrett & Hough, 2013). Temporal changes in patterns of sexual dimorphism during plant life‐cycles can result from the timing of the different reproductive roles of the sexes (Delph, 1999; Hesse & Pannell, 2011; Sánchez Vilas & Pannell, 2011). For species with wind‐mediated pollen and seed dispersal, males optimise pollen dispersal during peak flowering, whereas females maximise pollen receipt during flowering and seed dispersal at reproductive maturity. Therefore, selection is likely to favour taller males at peak flowering and taller females at reproductive maturity, leading to temporal changes in sexual dimorphism for plant height (Pickup & Barrett, 2012). These dynamics highlight the value of measuring sexual dimorphism at different life‐cycle stages to capture the complexity of sex‐specific roles (Harris & Pannell, 2008; Hesse & Pannell, 2011; Sánchez Vilas & Pannell, 2011; Teitel *et al*., 2016).

Differences in sex‐specific trait optima among populations can reflect the balance between sexual and natural selection mediated by local ecological conditions (Lande, 1980). Sex‐specific differences in reproductive costs and allocation trade‐offs (Lloyd & Webb, 1977; Delph, 1999; Obeso, 2002) may result in differential responses of each sex to environmental gradients (e.g. rainfall, temperature, Delph *et al*., 2011a), thereby contributing to heterogeneity in patterns of dimorphism, which in some cases may result in geographical clines. Although sex‐specific plasticity in trait expression across environmental conditions has been reported in several dioecious plant species (Delph & Bell, 2008; Teitel *et al*., 2016), among‐population variation in sexual dimorphism has not been investigated in detail (but see Delph *et al*., 2002). Moreover, to date, no studies have used common gardens to examine population‐level variation in sexual dimorphism in relation to the environment of source populations.

For wind‐pollinated species, demographic factors including population size and plant density are likely to influence sexual selection by mediating the degree of male‐male competition (Steven & Waller, 2007; Stehlik *et al*., 2008; Friedman & Barrett, 2009; Tonnabel *et al*., 2019). Biased sex ratios can also influence the strength of sexual selection by varying the degree of pollen competition (Compagnoni *et al*., 2017), with less competition expected in populations with female‐biased sex ratios. Disentangling the relative importance of these processes requires investigation of patterns of sexual dimorphism in different environmental and demographic contexts and for multiple populations spanning a species’ geographic range, an approach we use here.

The evolution of sexual dimorphism results from the interplay between sex‐specific selection and the underlying genetic architecture of traits (Delph *et al*., 2002, 2010; Ashman, 2003; Weller *et al*., 2006). Strong intersex genetic correlations may constrain the evolution of sexual dimorphism (Lande, 1980; Meagher, 1992) and intertrait correlations can lead to the evolution of sexual dimorphism for traits that are not directly under selection (Delph *et al*., 2002). Finally, sex‐specific differences in correlation among clusters of traits may impose constraints or result in trait coevolution in a sex‐specific manner (Meagher, 1992; Delph *et al*., 2002, 2005). These complexities highlight the importance of sex‐specific intertrait and intersex correlations for predicting responses to selection and the evolution of sexual dimorphism.

The more recent evolution of dioecy and sex chromosomes in angiosperms (Charlesworth, 2002; Ming *et al*., 2011) than in most animals provides the potential to examine sexual dimorphism in relation to sex chromosome variation (Govindarajulu *et al*., 2013; Charlesworth, 2018). In dioecious *Rumex* (Polygonaceae), sex chromosome systems vary both within and between species (Navajas‐Pérez *et al*., 2005; Cuñado *et al*., 2007). *Rumex hastatulus* possesses two distinct karyotype races with different sex chromosomes (Texas race XY; North Carolina XY_1_Y_2_; Smith, 1963; Bartkowiak, 1971). Karyotype differences may affect divergence between male and female phenotypes in two ways. First, sex‐linked genes may contribute disproportionately to the patterns of phenotypic and genetic differentiation between the races (Beaudry *et al*., 2019). Second, sex chromosomes may influence patterns of sex‐specific adaptation and intersex correlations through differences in dosage compensation and/or unequal transmission between the sexes (Rice, 1984; Dean & Mank, 2014). This species therefore provides a unique opportunity to determine whether sex chromosome variation contributes to patterns of sexual dimorphism.

Here, we examine spatial and temporal variation in genetically based sexual dimorphism in *R. hastatulus*. We measured quantitative traits under uniform glasshouse conditions across three life‐cycle stages corresponding to prereproduction, peak flowering and reproductive maturity in 30 populations sampled from across the geographical range of the species, including the two chromosome races. Specifically, we asked the following questions: (1) does sexual dimorphism in reproductive and vegetative traits vary among life‐cycle stages in relation to the different reproductive roles of females and males? (2) Does sexual dimorphism vary among populations across its geographic range and between chromosome races? (3) Can demographic, geographical and bioclimatic variables explain among‐population variation in sexual dimorphism? Having established the overall patterns of sexual dimorphism in *R. hastatulus* we then investigated trait correlations within and between the sexes to ask if intra‐ and intersex correlations vary across the life‐cycle for reproductive and vegetative traits. Our findings demonstrate that patterns of sexual dimorphism vary across the life‐cycle associated with differences between the sexes in reproductive roles, but also vary geographically, likely because of sex‐specific responses to bioclimatic parameters.

## Materials and Methods

### Study species and population sampling

*Rumex hastatulus* (Polygonaceae) is a largely annual coloniser of open sites distributed across the southern regions of the USA from Texas to North Carolina and Florida. Both pollen and seed of *R. hastatulus* are wind dispersed. The species is cytologically complex with two main chromosome races (Smith, 1963); the North Carolina karyotype (females = XX, 2*n* = 8; males = XY_1_Y_2_, 2*n* = 9) and the Texas karyotype (females XX, males XY, 2*n* = 10). Populations of the Texas race are distributed across four states: Texas (TX), Oklahoma (OK), Arkansas (AK) and Louisiana (LA), whereas populations of the North Carolina race occur in North Carolina (NC), South Carolina (SC), Georgia (GA), Alabama (AL) and Florida (FL).

To examine geographical variation in sexual dimorphism we sampled 30 populations of *R. hastatulus*, including 15 from each chromosome race (Supporting Information Fig. S1). The populations represent a subsample of 46 populations previously used to examine sex‐ratio variation (see Pickup & Barrett, 2013). The 30 populations were chosen based on two criteria: (1) seed was available from at least 20 maternal plants and (2) to span the observed variation in population size (TX race range = 66 to *c*. 2 000 000; NC race range = 10 to *c*. 556 000) and plant density (TX race range = 0.21–122.4 plants m^−2^; NC race range = 0.04–34.3 plants m^−2^) within each chromosome race. For each population, open‐pollinated seed families were collected from randomly chosen females along transects (for further details see Pickup & Barrett, 2013).

### Experimental design and traits measured

In June 2010, we germinated six seeds from 15 randomly chosen maternal plants (90 seeds per population) from each of the 30 populations (2700 seeds in total). Seeds were soaked in water for 24 h at 4°C and transferred to moist filter paper in Petri dishes in a growth cabinet maintained at 20°C for 12 h and 10°C for 12 h with continuous light. After *c*. 14 d we randomly chose and transplanted 60 seedlings (four from each of the 15 families) per population individually to 5‐cm pots containing Pro‐Mix BX (peat moss, vermiculate and perlite) and NPK fertiliser (20 : 20 : 20) and these were grown in a glasshouse at 20–24°C. Due to maternal variation in germination, 48–64 seedlings were planted per population (mean = 59.7; average of 3.4 seedlings per family, all with male and female representation, with sex determined at flowering). The 1792 seedlings were positioned in a complete randomised block design in the glasshouse.

To examine variation in sexual dimorphism among populations and during the life‐cycle, we measured traits at three growth stages: prereproduction (2 wk), peak flowering (4 wk) and reproductive maturity (8 wk) from planting date. (1) Plant height (vertical height from the pot surface to the tallest point on the plant), (2) number of leaves and (3) leaf size were measured at each life‐cycle stage. At 4 wk and 8 wk, we measured several reproductive traits: (4) flowering (presence of flowers in anthesis), (5) number of stems, (6) number of flowering stems, (7) number of inflorescences, and (8) length of three representative inflorescences (as a surrogate for the number of flowers per inflorescence, see Pickup & Barrett, 2013). We additionally calculated: (9) flowering as a binary variable (yes/no), (10) proportion of flowering stems for those individuals flowering and (11) an estimate of total flower number, by multiplying the number of inflorescences (7) by average inflorescence length (8). Sex (male, female), was determined at wk 4 or 8 by flower morphology. Nonflowering individuals could not be sexed and were therefore not considered in the analyses. At reproductive maturity (8 wk) we harvested plants and separated the above‐ground biomass into (12) vegetative biomass (including rosette leaves, stem leaves and stems), and (13) reproductive biomass (including inflorescences, and seeds and fruit for females). Total biomass (14) was represented as the sum of vegetative and reproductive biomass. We obtained dried weights for each biomass component by drying samples at 55°C for 3 d before weighing them on a four decimal place gram balance.

### Statistical analysis

To examine if sexual dimorphism in morphological and reproductive traits varied with chromosome race, population and life‐cycle stage we used generalised linear mixed models (GLMMs, function ‘glmer’ of the R package ‘lme4’). For overall models of sexual dimorphism, sex, chromosome race (or population) and life‐cycle stage were included as fixed effects, and maternal parent (nested within population) as a random effect. In models analysing temporal variation, life‐cycle stage was additionally included as a fixed effect, and individual as a random effect to correct for nonindependence of observations across time points. However, given a significant interaction between life‐cycle stage and sex (particularly strong between the first time point and the other two – see Table S1), the effect of chromosome race and population were examined using models for each life‐cycle stage separately. In sex × chromosome race models, population was included as a random effect. The probability distribution and link function used for each specific model were chosen by considering the: (1) nature of the response variable, (2) relation between the mean and variance of the response variable, and (3) quantile‐to‐quantile plots of the response variable vs data generated under different candidate distributions. Model choice was based on: (1) Akaike information criterion (AIC) values, (2) normal independent and identically distributed residuals, and (3) low correlation between residuals and fitted values and high correlation between predicted and observed values. These are standard criteria to decide on modeling strategy when using GLMMs (Bolker *et al*., 2009). We checked for overdispersion in Poisson and binomial models, and these were resolved by including ‘individual’ as a random effect. We determined the overall effects and significance of the fixed factors using type 2 ANOVA. When there was a significant interaction between sex and chromosome race, we used chromosome race‐specific models to examine overall sexual dimorphism and among‐population variation. For each model, we used the ‘predictmeans’ (R package) to obtain predicted means (conditional on all other sources of variation included in the models) and 95% confidence intervals, which were used for display and posterior analyses. These analyses, and all subsequent statistical analyses, were performed using R v.3.4.4 (R Core Team 2018).

#### Percent sexual dimorphism

To evaluate patterns of sexual dimorphism we calculated percent sexual dimorphism (%SD) as 100 × (mean_F_ − mean_M_)/mean_M_, where mean_M_ and mean_F_ are the predicted means for males and females, respectively (see Delph *et al*., 2002), for each trait and time point, both within and among populations and chromosome races. Positive values indicate female‐biased sexual dimorphism, while negative values indicate male‐biased sexual dimorphism. We calculated the confidence intervals as √(min_F_^2^ + min_M_^2^), where min_F_ and min_M_ are the lower (or upper) boundary of the 95% confidence interval for males and females given by the ‘predictmeans’ function from the GLM models.

#### Variation in sexual dimorphism among populations along demographic, geographical and bioclimatic gradients

We used multiple linear regression to examine if demographic (population size, population density and sex ratio), geographical (elevation, latitude and longitude) and bioclimatic parameters could explain among‐population variation in percent sexual dimorphism. We measured demographic parameters in the field in May−June 2009. For populations with <200 individuals, total population size, density (plants m^−2^) and sex ratio as no. females/(no. females + no. males) were obtained by direct counts. For large populations (>200 individuals), demographic parameters were estimated from stratified quadrats along four randomly positioned transects (see Pickup & Barrett, 2013 for full details on sampling and the data obtained). For each population, we obtained data for 19 bioclimatic variables (see Fig. S4a) from WorldClim v.1.4. (Hijmans *et al*., 2005), which provides high‐resolution (*c*. 1 km) interpolated climate surfaces based on monthly averages from 1960 to 1990.

To examine if these parameters explained significant variation in percent sexual dimorphism we used separate models for: (1) demographic, (2) geographical, and (3) bioclimatic variables. For the set of models examining bioclimatic variables, we first reduced the number of predictors to ensure their independence (determined using Spearman rank correlations, *r*_s_, Fig. S4a), and also that the predictors recapitulated the observed geographical clines by examining the two‐first principal components of the selected bioclimatic variables in comparison to all variables (Fig. S4b). In all models and for all traits and life‐cycle stages, explanatory variables were added sequentially to models of increasing complexity and ANOVA was used for model selection to identify the variable(s) that best explained differences in sexual dimorphism. For each model, we tested for homogeneity of residuals using the Shapiro−Wilk normality test. To facilitate interpretation of the contribution of bioclimatic variables to variation in sexual dimorphism, we regressed sex‐specific means on these variables for traits where multiple regressions were significant (see later, Table S3). We also visualised the relations between each parameter and sexual dimorphism to assess the direction of the correlations and whether heterogeneity in sexual dimorphism scaled with each predictor using funnel plots.

#### Intersex and intertrait correlations

We estimated intersex and intertrait correlations at wk 4 and 8 separately as Spearman rank correlation coefficients (*r*_s_) across predicted means including all populations. Given that our experiment was designed to maximise the number of populations sampled, there were too few individuals within families in each population to enable maternal variation to be taken into account in the calculation of population‐level correlations. To account for the potential effect of confounding variables on trait correlations, partial intersex and intertrait correlations were additionally calculated using ‘pcor.test’ function from the ‘ppcor’ R package with the Spearman method, by controlling for the predicted population means of the rest of the trait values (and averaged across sexes for the intersex partial correlations). We determined whether pairwise intertrait correlations differed significantly between the sexes via bootstrapping: we calculated sex‐specific 95% confidence intervals for correlations between all pairs of traits by selecting 25 out of all populations 1000 times. If the sex‐specific confidence intervals did not overlap we concluded that there was a significant difference in intertrait correlations. Pairwise correlations between absolute values of percent sexual dimorphism and intersex correlation were computed as Spearman rank correlation coefficients. For all analyses, *P*‐values indicate probability that *r*_s_ = 0.

## Results

### Sexual dimorphism in vegetative traits

**Figure 1:**
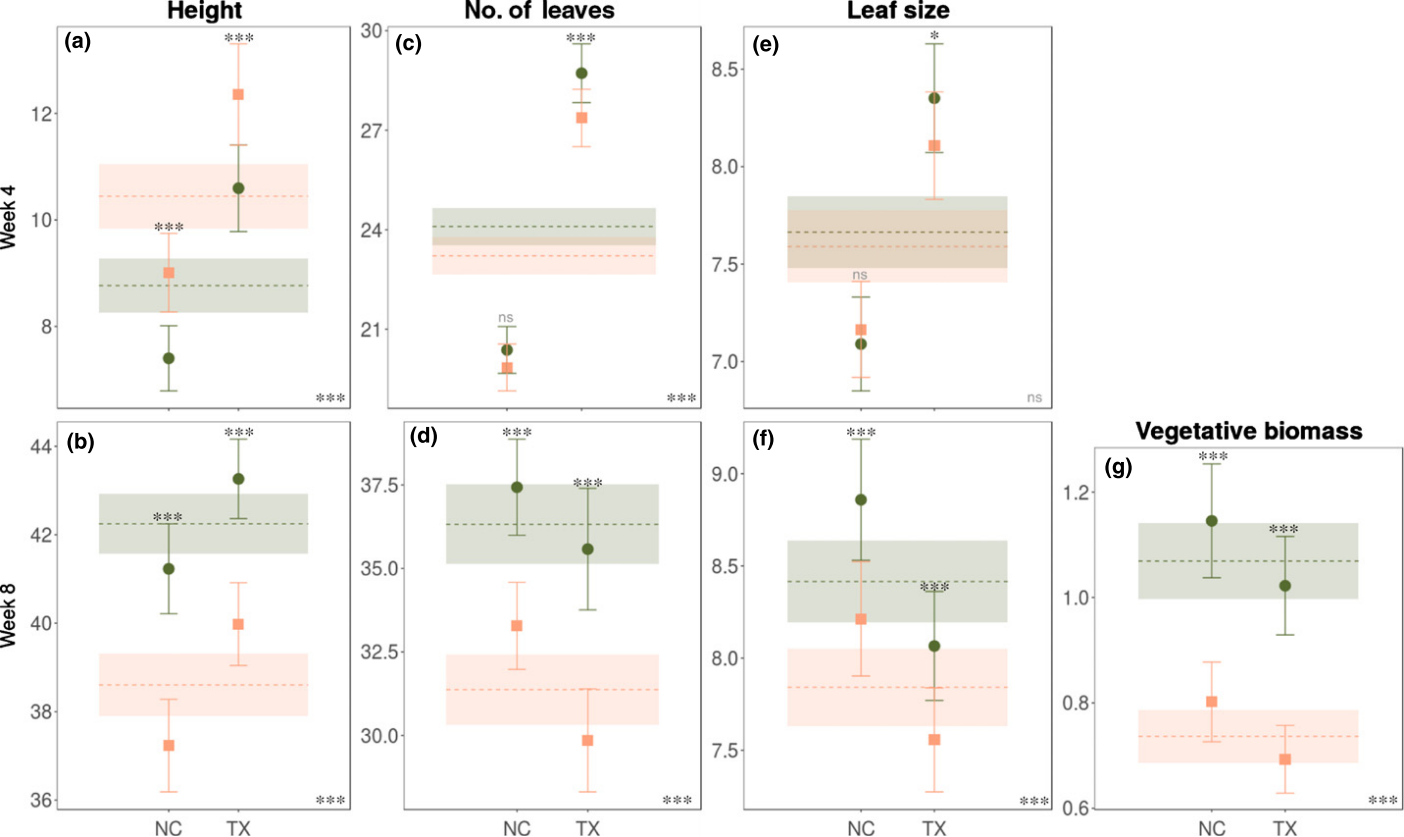
Sexual dimorphism of vegetative traits at 4 and 8 wk in *Rumex hastatulus*. Predicted means and 95% confidence intervals at two time points (4 and 8 wk) for males (orange squares) and females (green circles) of the Texas (TX) and North Carolina (NC) chromosome races (individual points) and overall values for each sex (dashed lines and colour shading). Traits measured at 4 and 8 wk were (a, b) height (cm), (c, d) number of leaves and (e, f) leaf size (cm), while (g) vegetative biomass (grams) was measured at harvest. The significance of sex differences for each chromosome race and across both races is indicated by stars above the individual bars and in the lower right corner of each plot, respectively. *, 0.01 < *P *<* *0.05; ***, *P *<* *0.001; ns, not statistically significant.

Sexual dimorphism in plant height changed significantly across the life‐cycle of *R. hastatulus*. There was no sexual dimorphism at wk 2 before flowering (Fig. S2a), but at wk 4 (peak flowering) males were significantly taller than females (%SD = −16.1; Figs 1a, 3 and Fig. S2b), and at wk 8 (reproductive maturity and seed dispersal) sexual dimorphism for height reversed, with females taller than males (%SD = 9.4; Figs 1b, 3 and Fig. S2c). The reversal in height was indicated by the significant interaction between sex and life‐cycle stage when the model included both 4 and 8 wk (Table cS1). These patterns of sexual dimorphism were consistent across populations and chromosome races, as indicated by the nonsignificant sex × population and sex × chromosome race interactions (Table S1). At wk 8, females produced more (%SD leaf number = 15.8) and larger leaves (%SD leaf size = 7.3; Figs 1d,f, 3) and this was reflected in female‐biased sexual dimorphism in vegetative biomass at harvest (%SD = 45.2; Figs 1g, 3). However, this pattern varied significantly among populations and between chromosome races (Table S1).

### Sexual dimorphism in reproductive traits

**Figure 2:**
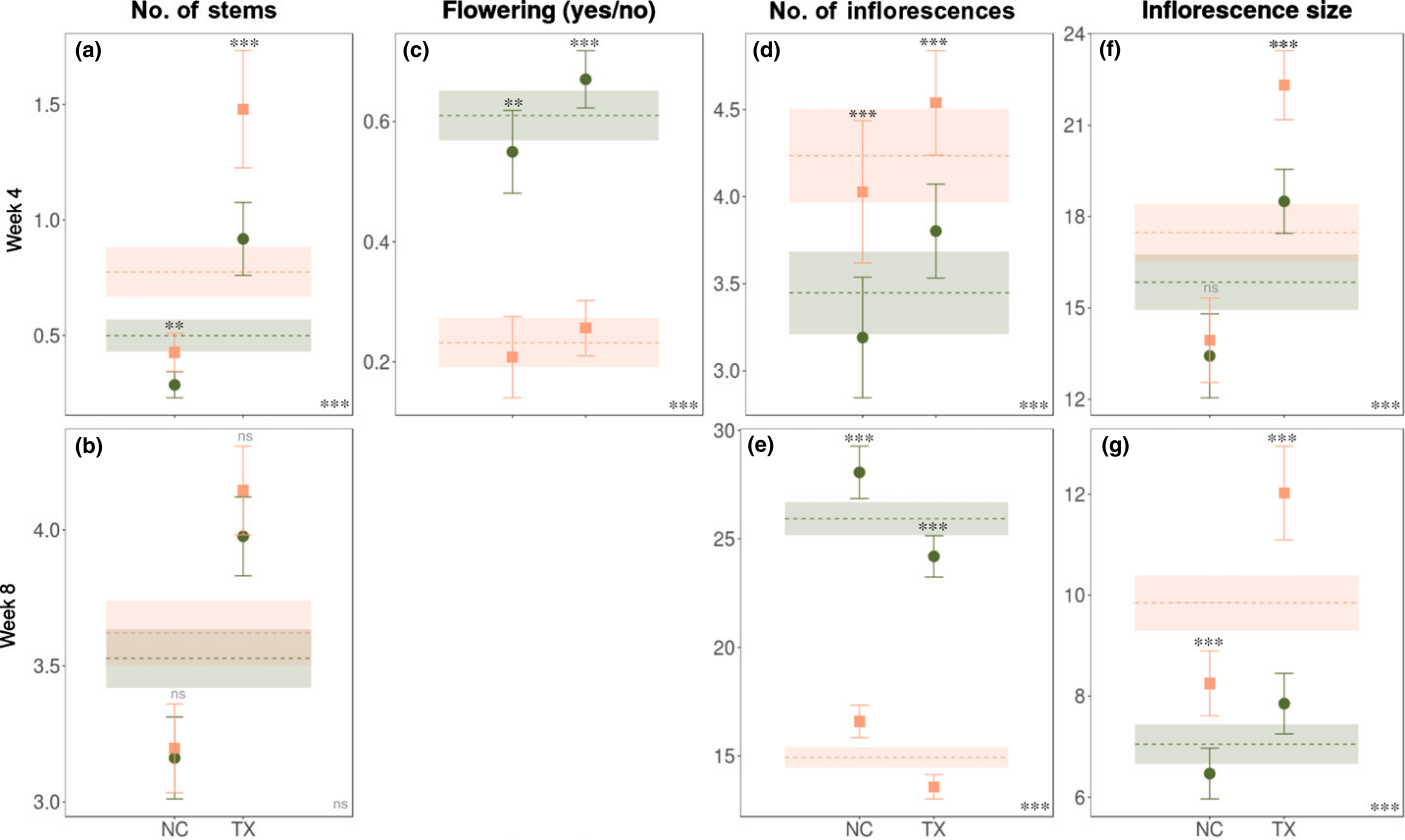
Sexual dimorphism of reproductive traits at 4 and 8 wk in *Rumex hastatulus*. Predicted means and 95% confidence intervals at two time points (4 and 8 wk) for males (orange squares) and females (green circles) of the Texas (TX) and North Carolina (NC) chromosome races (individual points) and overall for each sex (dashed lines and colour shading). Traits measured: (a, b) number of stems, (c) presence of flowering (yes/no) at wk 4 (at wk 8 all individuals are flowering, and (d, e) number of inflorescences and (f, g) inflorescence size (mm) for those individuals flowering. The significance of sex differences for each chromosome race and across both races is indicated by stars above the individual bars and in the lower right corner of each plot, respectively. **, 0.001 < *P *<* *0.01; ***, *P *<* *0.001; ns, not statistically significant.

**Figure 3:**
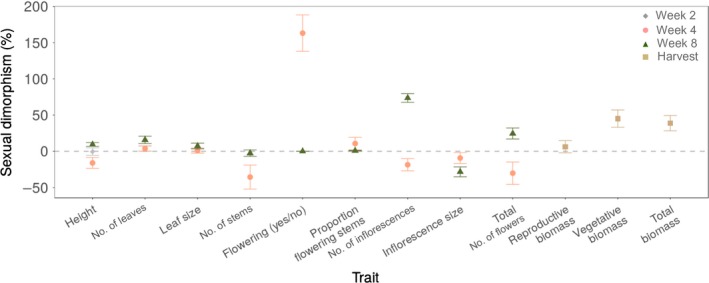
Percent of sexual dimorphism (%SD) per trait and at different life‐cycle stages for *Rumex hastatulus*. Percent sexual dimorphism was calculated as 100 × (mean_F_ − mean_M_)/mean_M_, where mean_M_ and mean_F_ are the predicted means for males and females respectively. Error bars represent 95% confidence intervals. Values above and below zero (dashed line) represent female‐biased and male‐biased sexual dimorphism, respectively.

At wk 4, even though males had more stems than females (Fig. 2a), the proportion of plants flowering was female biased, and this was the most sexually dimorphic trait overall (%SD = 163.2; Figs 2c, 3), suggesting that males may delay flowering and invest in stem growth. Indeed, we found a significant positive correlation (*r*_s_ = 0.81, *P *<* *0.0001) between height and total flower number in males at this life‐cycle stage. Among those individuals flowering at wk 4, males produced more and larger inflorescences than females (Fig. 2d,f). At reproductive maturity (wk 8), both sexes had equal numbers of stems (Fig. 2b), all of which had flowered. At wk 8 females produced more inflorescences (Fig. 2e), whereas in males inflorescences were larger (Fig. 2g). This difference resulted in a temporal reversal in sexual dimorphism from male biased at wk 4 to female biased at wk 8 for inflorescence number (%SD wk 4 = −18.6, %SD wk 8 = 73.7) and total flower number (inflorescence number × size, %SD wk 4 = −30.2, %SD wk 8 = 24.6; Fig. 3).

### Variation in sexual dimorphism among chromosome races and populations

There were no clear differences in the degree of sexual dimorphism between the Texas and North Carolina chromosome races of *R. hastatulus* (Figs 1, 2). For some traits (height, leaf size and number, amount of stems and inflorescence size and number), however, we found significantly higher sexual dimorphism in the Texas race compared with the North Carolina race at wk 4 (peak flowering) but not at wk 8 (Figs 1, 2; Table S1). This may reflect a developmental difference between the chromosome races, which could contribute to the earlier onset of sexual dimorphism in populations of the Texas karyotype. However, there was large among‐population variation in sexual dimorphism for many traits and across different life‐cycle stages (Fig. S3; see also sex × population interactions in (Table S1) As an example, Fig. S2 shows interpopulation variability for height across time points. Traits with a significant sex × population interaction were height at wk 2, inflorescence number and size at wk 4, number of leaves, proportion of flowering stems and inflorescence number and size at wk 8, and reproductive biomass at harvest (see Table S1).

**Figure 4:**
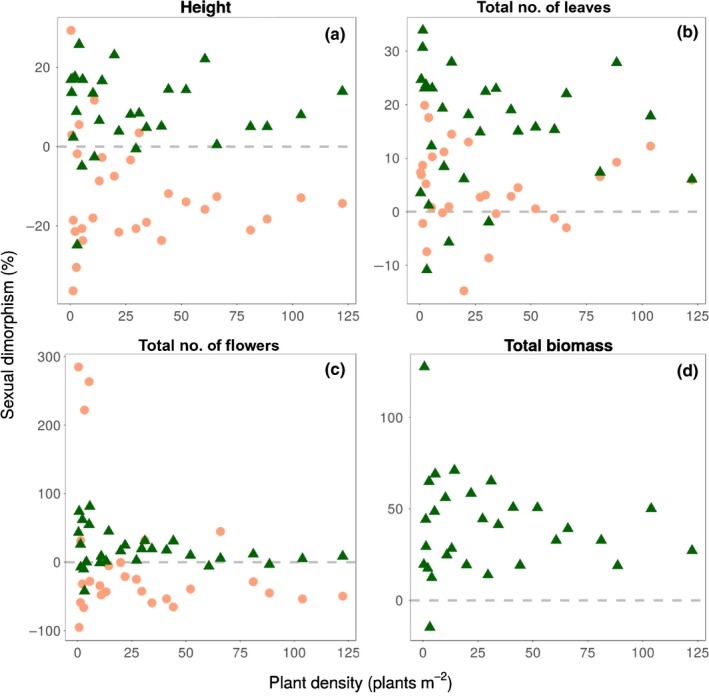
The relationship between percent sexual dimorphism (%SD) and mean plant density (plants m^−2^) for 29 populations of *Rumex hastatulus* for: (a) plant height (cm), (b) number of leaves, and (c) total flower number (number of inflorescences × inflorescence size) at wk 4 (orange circles) and wk 8 (green triangles). Total biomass (d) was measured at harvest. Values above and below zero (dashed line) represent female‐biased and male‐biased sexual dimorphism, respectively.

Next, we assessed whether the observed genetically based sexual dimorphism under glasshouse conditions could be explained by demographic, geographical and environmental variables of the population of origin. Population size, density and sex ratio did not explain significant variation in the degree of sexual dimorphism. Only sexual dimorphism in inflorescence size at wk 8 decreased with plant density (%SD inflorescence size, wk 8 = −23.124–0.167Density; *R* ^2^ = 0.28, *P *=* *0.0017). However, greater variability in sexual dimorphism for vegetative and reproductive traits at both wk 4 and 8 was evident in less dense populations (Fig. 4). Among populations, both male‐ and female‐biased sexual dimorphism was evident at low density for height, total flower number, number of leaves and biomass, but at higher density sexual dimorphism was more consistent in the direction of bias, which varied among traits (Fig. 4).

**Figure 5:**
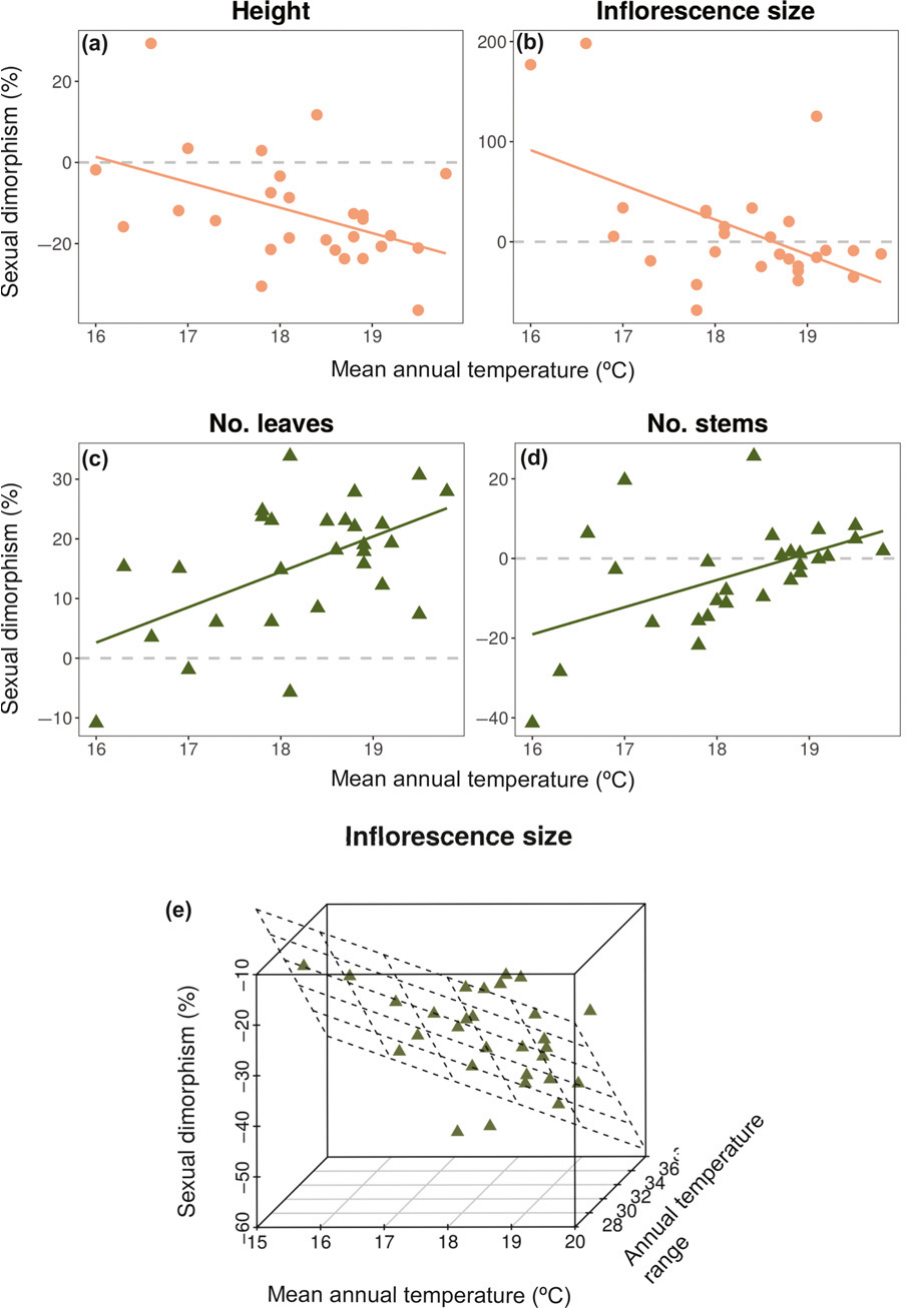
Patterns of sexual dimorphism along temperature gradients for populations of *Rumex hastatulus*. Per cent sexual dimorphism (%SD) among populations for different vegetative and reproductive traits at wk 4 (orange circles) and wk 8 (green triangles) plotted against mean annual temperature and annual temperature range and total annual precipitation (see Supporting Information Fig. S4 for more details on temperature variables). (a) Height (wk 4) = 99.40 −0.61bio1 (*R* ^2^ = 0.24, *P *=* *0.004); (b) inflorescence size (wk 4) = 647.71 −3.48bio1 (*R* ^2^ = 0.25, *P *=* *0.005); (c) number of leaves (wk 8) = −86.17 + 0.56bio1 (*R* ^2^ = 0.31, *P *=* *0.001); (d) number of stems (wk 8) = −103.10 + 0.55bio1 (*R* ^2^ = 0.19, *P *=* *0.011); (e) inflorescence size (wk 8) = 179.61 −0.45bio1 −0.39bio7 (*R* ^2^ = 0.43, *P *=* *0.0003).

Geographical parameters (longitude, latitude and elevation) of the population of origin explained between 14–35% of the interpopulation variation in sexual dimorphism for several vegetative and flowering traits in the glasshouse (see Table S2). Given that these patterns are likely to reflect underlying bioclimatic variation along geographical clines, we examined sexual dimorphism in relation to three bioclimatic parameters of the source populations: total annual precipitation, annual mean temperature and annual temperature range (based on reduced dimensionality of 19 WorldClim bioclimatic parameters, see Fig. S4 and the Materials and Methods section for details), which were indeed correlated with longitude, latitude and elevation (Fig. S4c). At wk 4, male‐biased sexual dimorphism in height increased with mean temperature (*R* ^2^ = 0.24, *P *=* *0.004; Fig. 5a), whereas for inflorescence size, the degree of sexual dimorphism changed from female biased to male biased with increasing mean temperature (*R* ^2^ = 0.25, *P *=* *0.0048; Fig. 5b). At wk 8, we found that mean temperature explained 31% (*P *=* *0.001) and 19% (*P *=* *0.011) of the variation in female‐biased sexual dimorphism in numbers of leaves and stems, respectively (Fig. 5c,d). At this life‐cycle stage, male‐biased dimorphism in inflorescence size was greater in populations with higher mean annual temperature and a smaller annual temperature range (*R* ^2^ = 0.43, *P *=* *0.002, Fig. 5e).

A more complex relationship between sexual dimorphism and bioclimatic variables was evident for numbers of stems and total flower number at 4 wk. For total flower number, sexual dimorphism was more male biased in populations with higher and more variable temperatures and greater annual precipitation, with these three variables explaining 34% of the variation in sexual dimorphism (total flower number = 3270.29 – 8.32mean temperature−4.09temperature range − 0.36precipitation; *P *=* *0.006). Sexual dimorphism in the number of stems was more male biased in populations with higher precipitation and annual variation in temperature, but lower average temperature (number of stems = 726.53 −1.12mean temperature −1.28temperature range −0.12precipitation; *R* ^2^ = 0.37, *P *=* *0.003). Sex‐specific regression of trait means on bioclimatic variables revealed that several such patterns probably resulted from differences between sexes in their sensitivity to environmental heterogeneity. For example, the increase in male‐biased sexual dimorphism in flowering at wk 4 at higher temperatures was likely to be due to males increasing flower production relative to females with increasing temperature (Table S3).

### Intersex and intertrait correlations

**Figure 6:**
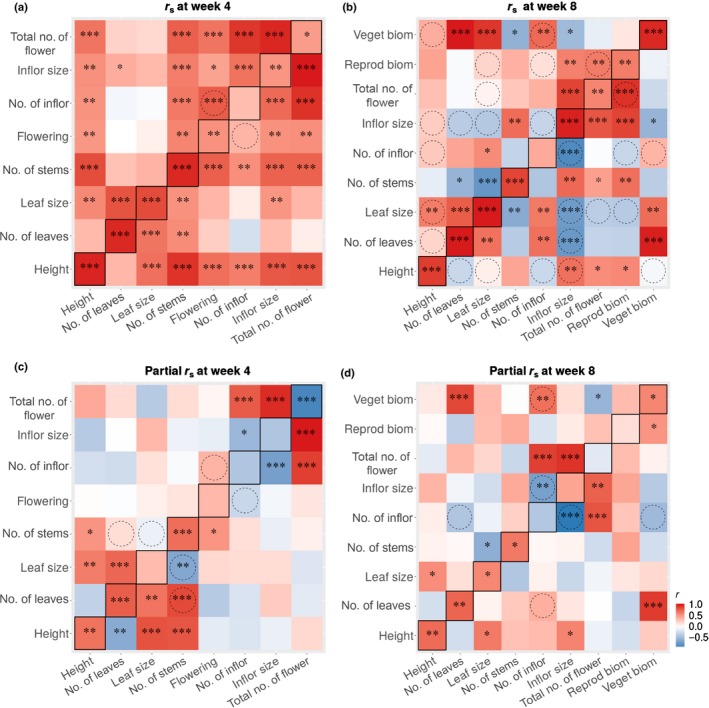
Intersex and intertrait raw and partial correlations for *Rumex hastatulus* using predicted population means at (a, c) 4 and (b, d) 8 wk. For each plot, above the diagonal are the intertrait correlations among females, below diagonal are the intertrait correlations among males and on‐diagonal are the intersex correlations for each trait. Reproductive and vegetative biomass were measured at harvest. Flowering (yes/no) is not included at wk 8 as all individuals flowered. For the raw correlations (a, b) values correspond to Spearman rank correlation on predicted population means. Partial correlations (c, d) control for potential confounding interactions with other traits. Colour indicates the direction of the correlation (red for positive and blue for negative), whereas the strength of the correlation is indicated by the colour bar. *, 0.01 < *P *<* *0.05; **, 0.001 < *P *<* *0.01; ***, *P *<* *0.001. Dashed circles denote significant differences between pairwise intertrait correlations between sexes, determined via bootstrapping (see the Materials and Methods section for more details).

We analysed intersex and intertrait correlations using the predicted means of populations at each life‐cycle stage separately, because the correlation among traits within each stage was higher than the within‐trait correlations across life‐cycle stages (data not shown). We found significant pairwise correlations between many traits, which in some cases differed between sexes (Fig. 6a,b). For example, height was positively correlated with leaf size in females and with inflorescence size in males, whereas inflorescence size was strongly negatively correlated with leaf production and inflorescence number in males but not females. These results are consistent with greater male investment in inflorescences, at the expense of vegetative traits, whereas females invest in both vegetative and reproductive structures.

We also detected significant temporal differences in intertrait correlations (Fig. 6a,b). Whereas sex differences and negative values of intertrait correlations were only apparent at wk 8, at wk 4 all intertrait correlations were positive and highly concordant between the sexes. This concordance at wk 4 is likely to reflect developmental variation, and that specific intertrait correlations may result from indirect interactions with other traits. Indeed, when examined via partial correlations (accounting for other traits as covariates), we found that trade‐offs between pairs of traits were more consistent across sexes and time points (Fig. 6c,d). For example, partial correlations showed that both sexes displayed a trade‐off between inflorescence size and number (which was stronger in males than females). Yet, for the uncorrected correlations, this was masked by indirect interactions with other traits at wk 4 for both sexes, and for females at wk 8.

We then explored the relationships between intersex correlation and the extent of sexual dimorphism. Interestingly, number of inflorescences, which had the lowest intersex correlation both at wk 4 and 8, also displayed temporal reversal in sexual dimorphism (Fig. 3). However, we found no significant covariation between intersex correlation and the extent of sexual dimorphism (*r*_s_ = −0.095, *P *=* *0.6745). Moreover, the most sexually dimorphic traits (flowering at wk 4 and inflorescence number at wk 8) had very similar correlations with other traits in both sexes.

## Discussion

We compared patterns of sexual dimorphism for reproductive and vegetative traits measured under uniform glasshouse conditions at three life‐cycle stages in 30 populations of dioecious wind‐pollinated *Rumex hastatulus*. Genetically based sexual dimorphism was evident for most traits and often changed during the life‐cycle, with a reversal of dimorphism between peak flowering and reproductive maturity for some traits (e.g. height, flowering). We detected no systematic sex differences between the chromosome races, but there was striking among‐population variation in sexual dimorphism, which was partially explained by bioclimatic variables along geographical clines. We now discuss how these patterns of sexual dimorphism relate to the reproductive roles of the sexes and their life‐cycle dynamics, and consider explanations for the among‐population variation in sexual dimorphism and how intertrait and intersex correlations may influence the evolution of sex differences.

### Temporal variation and reproductive roles of the sexes

Sexual dimorphism of reproductive and vegetative traits is widespread among dioecious plant species (Delph, 1999; Barrett & Hough, 2013), reflecting the different reproductive roles of females and males (Lloyd & Webb, 1977), sex‐specific trade‐offs in resource use (Moore & Pannell, 2011) and interactions with underlying intersex genetic correlations (Delph *et al*., 2002, 2010, 2011b). The patterns of sexual dimorphism we observed in *R. hastatulus* are consistent with temporal differences in the reproductive roles of the sexes. For example, males were taller at peak flowering, which is likely to facilitate wind‐mediated pollen dispersal in males and pollen receipt in females (Okubo & Levin, 1989; Friedman & Barrett, 2009), whereas females were taller at reproductive maturity, which is likely to increase the dispersal distance of wind‐dispersed seeds (Tackenberg *et al*., 2003; Soons *et al*., 2004; Thomson *et al*., 2011; Bullock *et al*., 2017; Fig. 1a,b). The benefit of increased seed dispersal distance in plants includes reduced sib‐competition and greater potential access to favourable microsites (Howe & Smallwood, 1982; Levin *et al*., 2003). Our finding of reversal in sexual dimorphism for height in *R. hastatulus*, extends previous results (Pickup & Barrett, 2012; Teitel *et al*., 2016), which involved many fewer populations of this species, and demonstrates that this pattern of height reversal is a fundamental feature of the growth strategy of this species.

Although we did not directly evaluate the reproductive success of males in relation to height, several lines of evidence suggested that male height reflected wind‐mediated sex‐specific selection (e.g. Tonnabel *et al*., 2019). First, we observed a significant positive relation between flowering onset and height in this sex, suggesting that males delay flowering to achieve increased stem elongation. This aspect may be particularly important in *R. hastatulus*, as this species occurs in monospecific stands in open habitats in which height – relative to conspecifics – is likely to promote more effective pollen dispersal (Niklas, 1985). Second, male‐biased dimorphism in height at peak flowering was consistent across most populations (Fig. S2b; nonsignificant sex × population interaction in Table S1). Significantly, the only populations where this was not evident were those at low plant density (Fig. 4a), where male−male competition may be less intense due to the positive relationship between plant density and stigmatic pollen loads in wind‐pollinated herbs (Steven & Waller, 2007; Friedman & Barrett, 2009; Hesse & Pannell, 2011), including *R. hastatulus* (M. Pickup, D.L. Field and S.C.H. Barrett, unpublished) and other *Rumex* species (Stehlik & Barrett, 2006; Stehlik *et al*., 2008). Accordingly, other vegetative and reproductive traits showed higher consistency in sexual dimorphism in denser populations (Fig. 4). Third, in males, height was correlated with inflorescence size (indicative of flower number; Fig. 6), suggesting that taller males have a higher reproductive investment and male siring success.

Wind‐mediated sexual selection also likely acts on females of *R. hastatulus*. Although shorter stature facilitates pollen receipt during peak flowering, taller females probably have increased seed dispersal at reproductive maturity, consistent with the temporal reversal of sexual dimorphism in height we observed (Fig. 1a,b). We also found evidence for sexual selection shaping patterns of sexual dimorphism in inflorescence traits. Overall, males produced fewer but taller and longer inflorescences (Fig. 2e,g), facilitating pollen dispersal, whereas females had more smaller inflorescences, which may optimise pollen receipt by distributing flowers over a larger portion of the air stream. We found greater vegetative biomass in females at reproductive maturity*,* a pattern reported in other herbaceous wind‐pollinated species (Korpelainen, 1992; Harris & Pannell, 2008; Hesse & Pannell, 2011), and a previous study of *R. hastatulus* (Teitel *et al*., 2016). Sex‐specific differences in vegetative investment in wind‐pollinated herbaceous plants have been explained by contrasting resource requirements, in that females need more carbon for seed and fruit production (Harris & Pannell, 2008; Teitel *et al*., 2016). We found greater female investment in leaves consistently across populations, especially at reproductive maturity (Fig. 1d,f,g). Female bias in vegetative biomass at reproductive maturity, but no difference between the sexes in number of stems (Fig. 2a), is likely to reflect greater investment in longer stems to support developing fruit and aid in their dispersal. Overall, we found higher sexual dimorphism in reproductive than vegetative traits, as reported for several other dioecious species (Delph *et al*., 2002; Barrett & Hough, 2013; Tonnabel *et al*., 2019; Fig. 3).

### Geographical variation in sexual dimorphism

Understanding the drivers of geographical variation in sexual dimorphism can provide insights into the importance of sexual selection, sex‐specific plasticity and genetic divergence of sex‐specific trait differences (Delph *et al*., 2002; Delph & Bell, 2008). Our common garden study revealed extensive genetically based among‐population variation in sexual dimorphism across the life‐cycle. We investigated several genetic and ecological correlates of this variation in an effort to provide insights into potential contributing factors. First, we predicted that genetic divergence at sex‐linked genes across the two karyotypic races (Beaudry *et al*., 2019) might contribute to sexual dimorphism because sex chromosomes may be enriched for variation influencing sex‐specific adaptations (Rice, 1984; Dean & Mank, 2014). However, we found no systematic sex differences between the chromosome races (Figs 1, 2). Significant among‐race differences in sexual dimorphism at wk 4 for some traits likely reflect a developmental difference associated with the earlier onset of dimorphism in populations of the Texas race.

Second, sex ratio might influence patterns of sex differences by mediating the degree of pollen competition, with greater competition expected in populations with more males. Although populations of *R. hastatulus* varied in degree of female‐bias (sex ratio: 0.54–0.68; Pickup & Barrett, 2013), sex ratio did not explain variation in sexual dimorphism. Third, differences in sexual dimorphism can also arise through a greater response of one sex than the other to environmental heterogeneity (Delph & Bell, 2008; Delph *et al*., 2011a). For example, sex‐specific responses to environmental variables have been reported in natural populations of *Salix* (Dudley, 2006), although it is difficult to disentangle plasticity from genetically based dimorphism by observing natural variation. We regressed genetically based sexual dimorphism and sex‐specific trait means on the bioclimatic parameters of source populations of *R. hastatulu*s to determine: (1) if sexual dimorphism varied across bioclimatic clines and, (2) whether these patterns are due to a greater response of one sex than the other. We found that a large proportion (up to 43%) of variation in sexual dimorphism in some traits was explained by bioclimatic variables, likely to be due to sex‐differential responses. Mean annual temperature is expected to provide more favourable growing conditions during the year. In our study, populations from sites with higher mean annual temperatures had greater male‐biased sexual dimorphism for plant height at peak flowering (Fig. 5a), perhaps due to a higher relative investment in stem growth in males than females at higher temperatures (Table S3). We also found a positive correlation between temperature and female‐biased sexual dimorphism for leaf production at reproductive maturity (Fig. 5c). In females, a slower rate of decline in mean leaf production with increasing annual temperature (see Table S3) suggested that they maintained a greater investment in leaves than males over this gradient, which was reflected in increased female‐biased dimorphism.

To our knowledge, our finding of correlations between geographical and bioclimatic variables and sexual dimorphism in *R. hastatulus*, provides the first evidence for clinal variation in sexual dimorphism in plants. Our results strongly suggest that both variation in sexual selection, mediated by intrasex competition and sex‐specific differences in resource allocation trade‐offs, modulated by bioclimatic variables, shape patterns of sexual dimorphism. Experimental manipulation of growing conditions could be used to further investigate these hypotheses and, combined with studies of selection gradients (see Delph & Herlihy, 2012), could provide more information on how these different factors interact in the evolution of sex differences.

### Phenotypic correlations and the evolution of sexual dimorphism

Our study examined intersex and intertrait correlations at the phenotypic level to understand how they interact with the evolution of sex differences. First, we found many pairwise trait correlations (Fig. 6a,b), yet, for many pairs of traits, the correlations changed in strength and direction when assessed using partial correlations conditioned on other traits (Fig. 6c,d). This observation suggests an extensive shared genetic basis across traits and that there is the potential for correlated evolution to drive sexual dimorphism (Lande, 1980; Delph *et al*., 2002, 2004a,2004b). We observed extensive sex‐specific differences in both the direction and magnitude of intertrait correlations for some traits, which may reflect sex differences in selective pressures and trait architecture (Ashman, 2003; Delph *et al*., 2010). For example, inflorescence size was negatively correlated with inflorescence number and leaf size in males, but not females, indicating that males invest in inflorescence size at the expense of the other traits, whereas females strike a compromise between reproductive and vegetative investment (Delph *et al*., 2005; Fig. 6). In general, males had more significant among‐trait correlations and trade‐offs than females, which is consistent with previous findings in *Silene latifolia* (Steven *et al*., 2007; Delph *et al*., 2010), *Ceratodon purpureous* (McDaniel, 2005) and *R. hastatulus* (Teitel *et al*., 2016). Importantly, in our study, negative correlations among traits only became evident at wk 8, indicating that wk 4 probably captured mostly developmental variation. This finding highlights the importance of examining intersex and intertrait correlations across the life‐cycle to capture functionally relevant patterns.

High intersex correlations in trait expression can limit the evolution of sex differences (Meagher, 1992; Ashman, 2003) and as a result constrain the evolution of sexual dimorphism (Poissant *et al*., 2010; Griffin *et al*., 2013). However, we found no association between intersex correlation and the extent of sexual dimorphism. The most dimorphic traits (flowering at wk 4 and inflorescence number at wk 8) had very similar correlations with other traits in both sexes (Fig. 6b). These results therefore suggested that, although intersex correlations contribute to patterns of sexual dimorphism, they are capable of evolving and are not inflexible constraints to the evolution of sex differences (Delph *et al*., 2011b).

Understanding the link between sex‐specific phenotypic variation and the different reproductive roles of the sexes has long intrigued evolutionary biologists. For plants, pollen and seed dispersal vectors can mediate the strength of sex‐specific selection, leading to trait changes in relation to the timing of the reproductive roles of males (pollen dispersal) and females (pollen receipt and seed dispersal). Similarly, interaction between environmental gradients and sex‐specific resource requirement may result in clinal variation in patterns of dimorphism. By examining geographical and temporal variation in sexual dimorphism, our study has provided novel insights into how sexual and natural selection contributed to sex‐phenotype variation in a wide‐ranging plant species.

## Author contributions

MP, DLF and SCHB conceived the study and designed the research experiment, MP and DLF undertook the experiment and data collection, GP, MP and DLF performed the data analyses and all authors contributed to writing of the manuscript. GP and MP contributed equally to this work.

## Supporting information

Please note: Wiley Blackwell are not responsible for the content or functionality of any Supporting Information supplied by the authors. Any queries (other than missing material) should be directed to the *New Phytologist* Central Office.

**Fig. S1** The geographic distribution of the 30 sampled populations of *Rumex hastatulus* across the southern USA.
**Fig. S2** Plant height across life‐cycle stages and populations of *Rumex hastatulus*.
**Fig. S3** Sexual dimorphism (%SD) among populations at wk 4 and wk 8 in *Rumex hastatulus*.
**Fig. S4** Bioclimatic variables across the geographical range of *Rumex hastatulus*.
**Table S1** Summary of univariate results for common glasshouse study of *Rumex hastatulus*.
**Table S2** Variation in sexual dimorphism across geographical gradients for populations of *Rumex hastatulus*.
**Table S3** Sexual dimorphism and sex‐specific trait mean variation along climatic gradients for populations of *Rumex hastatulus*.

## Data Availability

Data are available in DRYAD (doi: https://doi.org/10.5061/dryad.n1701c9).

## References

[nph16050-bib-0001] Andersson MB . 1994 Sexual selection. Princeton, NJ, USA: Princeton University Press.

[nph16050-bib-0002] Ashman T‐L . 2003 Constraints on the evolution of males and sexual dimorphism: field estimates of genetic architecture of reproductive traits in three populations of gynodioecious *Fragaria virginiana* . Evolution 57: 2012–2025. 14575323 10.1111/j.0014-3820.2003.tb00381.x

[nph16050-bib-0003] Barrett SCH , Hough J . 2013 Sexual dimorphism in flowering plants. Journal of Experimental Botany 64: 67–82. 23183260 10.1093/jxb/ers308

[nph16050-bib-0004] Bartkowiak E . 1971 Mechanism of sex determination in *Rumex hastatulus* Baldw. Theoretical and Applied Genetics 41: 320–326. 24430447 10.1007/BF00577104

[nph16050-bib-0005] Beaudry FEG , Barrett SCH , Wright SI . 2019 Ancestral and neo‐sex chromosomes contribute to population divergence in a dioecious plant. bioRxiv. doi: 10.1101/550962. 31808547

[nph16050-bib-0006] Bolker BM , Brooks ME , Clark CJ , Geange SW , Poulsen JR , Stevens MHH , White J‐SS . 2009 Generalized linear mixed models: a practical guide for ecology and evolution. Trends in Ecology & Evolution 24: 127–135. 19185386 10.1016/j.tree.2008.10.008

[nph16050-bib-0007] Bullock JM , González LM , Tamme R , Götzenberger L , White SM , Pärtel M , Hooftman DAP . 2017 A synthesis of empirical plant dispersal kernels. Journal of Ecology 105: 6–19.

[nph16050-bib-0008] Charlesworth D . 2002 Plant sex determination and sex chromosomes. Heredity 88: 94–101. 11932767 10.1038/sj.hdy.6800016

[nph16050-bib-0009] Charlesworth D . 2018 Does sexual dimorphism in plants promote sex chromosome evolution? Environmental and Experimental Botany 146: 5–12.

[nph16050-bib-0010] Compagnoni A , Steigman K , Miller TEX . 2017 Can't live with them, can't live without them? Balancing mating and competition in two‐sex populations. Proceedings of the Royal Society of London. Series B: Biological Sciences 284: pii: 20171999. 10.1098/rspb.2017.1999 PMC5666111 29070729

[nph16050-bib-0011] Cuñado N , Navajas‐Pérez R , de la Herrán R , Ruiz Rejón C , Ruiz Rejón M , Santos JL , Garrido‐Ramos MA . 2007 The evolution of sex chromosomes in the genus *Rumex* (Polygonaceae): identification of a new species with heteromorphic sex chromosomes. Chromosome Research 15: 825–833. 17899410 10.1007/s10577-007-1166-6

[nph16050-bib-0012] Darwin C . 1871 The descent of man and selection in relation to sex. London, UK: John Murray.

[nph16050-bib-0013] Dean R , Mank JE . 2014 The role of sex chromosomes in sexual dimorphism: discordance between molecular and phenotypic data. Journal of Evolutionary Biology 27: 1443–1453. 25105198 10.1111/jeb.12345

[nph16050-bib-0014] Delph LF . 1999 Sexual dimorphism in life history In: Geber MA, Dawson TE, Delph LF, eds. Gender and sexual dimorphism in flowering plants. Berlin, Heidelberg, Germany: Springer, 149–173.

[nph16050-bib-0015] Delph LF , Andicoechea J , Steven JC , Herlihy CR , Scarpino SV , Bell DL . 2011a Environment‐dependent intralocus sexual conflict in a dioecious plant. New Phytologist 192: 542–552. 21726233 10.1111/j.1469-8137.2011.03811.x

[nph16050-bib-0016] Delph LF , Arntz AM , Scotti‐Saintagne C , Scotti I . 2010 The genomic architecture of sexual dimorphism in the dioecious plant *Silene latifolia* . Evolution 64: 2873–2886. 20550575 10.1111/j.1558-5646.2010.01048.x

[nph16050-bib-0017] Delph LF , Bell DL . 2008 A test of the differential‐plasticity hypothesis for variation in the degree of sexual dimorphism in *Silene latifolia* . Evolutionary Ecology Research 10: 61–75.

[nph16050-bib-0018] Delph LF , Frey FM , Steven JC , Gehring JL . 2004a Investigating the independent evolution of the size of floral organs via G‐matrix estimation and artificial selection. Evolution & Development 6: 438–448. 15509226 10.1111/j.1525-142X.2004.04052.x

[nph16050-bib-0019] Delph LF , Gehring JL , Arntz AM , Levri M , Frey FM . 2005 Genetic correlations with floral display lead to sexual dimorphism in the cost of reproduction. American Naturalist 166(Suppl 4): S31–S41. 10.1086/444597 16224710

[nph16050-bib-0020] Delph LF , Gehring JL , Frey FM , Arntz AM , Levri M . 2004b Genetic constraints on floral evolution in a sexually dimorphic plant revealed by artificial selection. Evolution 58: 1936–1946. 15521453 10.1111/j.0014-3820.2004.tb00481.x

[nph16050-bib-0021] Delph LF , Herlihy CR . 2012 Sexual, fecundity, and viability selection on flower size and number in a sexually dimorphic plant. Evolution 66: 1154–1166. 22486695 10.1111/j.1558-5646.2011.01510.x

[nph16050-bib-0022] Delph LF , Knapczyk FN , Taylor DR . 2002 Among‐population variation and correlations in sexually dimorphic traits of *Silene latifolia* . Journal of Evolutionary Biology 15: 1011–1020.

[nph16050-bib-0023] Delph LF , Steven JC , Anderson IA , Herlihy CR , Brodie ED III . 2011b Elimination of a genetic correlation between the sexes via artificial correlational selection. Evolution 65: 2872–2880. 21967428 10.1111/j.1558-5646.2011.01350.x

[nph16050-bib-0024] Dudley LS . 2006 Ecological correlates of secondary sexual dimorphism in *Salix glauca* (Salicaceae). American Journal of Botany 93: 1775–1783. 21642123 10.3732/ajb.93.12.1775

[nph16050-bib-0025] Eppley SM , Pannell JR . 2007 Density‐dependent self‐fertilization and male versus hermaphrodite siring success in an androdioecious plant. Evolution 61: 2349–2359. 17711472 10.1111/j.1558-5646.2007.00195.x

[nph16050-bib-0026] Friedman J , Barrett SCH . 2009 Wind of change: new insights on the ecology and evolution of pollination and mating in wind‐pollinated plants. Annals of Botany 103: 1515–1527. 19218583 10.1093/aob/mcp035 PMC2701749

[nph16050-bib-0027] Friedman J , Barrett SCH . 2011 The evolution of ovule number and flower size in wind‐pollinated plants. American Naturalist 177: 246–257. 10.1086/657954 21460560

[nph16050-bib-0028] Geber MA , Dawson TE , Delph LF . 1999 Gender and sexual dimorphism in flowering plants. Berlin, Heidelberg, Germany: Springer.

[nph16050-bib-0029] Govindarajulu R , Liston A , Ashman T‐L . 2013 Sex‐determining chromosomes and sexual dimorphism: insights from genetic mapping of sex expression in a natural hybrid *Fragaria × ananassa* subsp. *cuneifolia* . Heredity 110: 430–438. 23169558 10.1038/hdy.2012.96 PMC3630810

[nph16050-bib-0030] Griffin RM , Dean R , Grace JL , Rydén P , Friberg U . 2013 The shared genome is a pervasive constraint on the evolution of sex‐biased gene expression. Molecular Biology and Evolution 30: 2168–2176. 23813981 10.1093/molbev/mst121

[nph16050-bib-0031] Harris MS , Pannell JR . 2008 Roots, shoots and reproduction: sexual dimorphism in size and costs of reproductive allocation in an annual herb. Proceedings of the Royal Society of London. Series B: Biological Sciences 275: 2595–2602. 18682371 10.1098/rspb.2008.0585 PMC2605799

[nph16050-bib-0032] Hesse E , Pannell JR . 2011 Sexual dimorphism in a dioecious population of the wind‐pollinated herb *Mercurialis annua*: the interactive effects of resource availability and competition. Annals of Botany 107: 1039–1045. 21385775 10.1093/aob/mcr046 PMC3080628

[nph16050-bib-0033] Hijmans RJ , Cameron SE , Parra JL , Jones PG , Jarvis A . 2005 Very high resolution interpolated climate surfaces for global land areas. International Journal of Climatology 25: 1965–1978.

[nph16050-bib-0034] Howe HF , Smallwood J . 1982 Ecology of seed dispersal. Annual Review of Ecology and Systematics 13: 201–228.

[nph16050-bib-0035] Korpelainen H . 1992 Patterns of resource allocation in male and female plants of *Rumex acetosa* and *R. acetosella* . Oecologia 89: 133–139. 28313405 10.1007/BF00319025

[nph16050-bib-0036] Lande R . 1980 Sexual dimorphism, sexual selection, and adaptation in polygenic characters. Evolution 34: 292–305. 28563426 10.1111/j.1558-5646.1980.tb04817.x

[nph16050-bib-0037] Levin SA , Muller‐Landau HC , Nathan R , Chave J . 2003 The ecology and evolution of seed dispersal: a theoretical perspective. Annual Review of Ecology, Evolution, and Systematics 34: 575–604.

[nph16050-bib-0038] Lloyd DG , Webb CJ . 1977 Secondary sex characters in plants. Botanical Review 43: 177–216.

[nph16050-bib-0039] McDaniel SF . 2005 Genetic correlations do not constrain the evolution of sexual dimorphism in the moss *Ceratodon purpureus* . Evolution 59: 2353–2361. 16396176

[nph16050-bib-0040] Meagher TR . 1992 The quantitative genetics of sexual dimorphism in *Silene latifolia* (Caryophyllaeae). I. Genetic variation. Evolution 46: 445–457. 28564016 10.1111/j.1558-5646.1992.tb02050.x

[nph16050-bib-0041] Ming R , Bendahmane A , Renner SS . 2011 Sex chromosomes in land plants. Annual Review of Plant Biology 62: 485–514. 10.1146/annurev-arplant-042110-103914 21526970

[nph16050-bib-0042] Moore JC , Pannell JR . 2011 Sexual selection in plants. Current Biology 21: R176–R182. 21377091 10.1016/j.cub.2010.12.035

[nph16050-bib-0043] Navajas‐Pérez R , de la Herrán R , López González G , Jamilena M , Lozano R , Ruiz Rejón C , Ruiz Rejón M , Garrido‐Ramos MA . 2005 The evolution of reproductive systems and sex‐determining mechanisms within *Rumex* (Polygonaceae) inferred from nuclear and chloroplastidial sequence data. Molecular Biology and Evolution 22: 1929–1939. 15944442 10.1093/molbev/msi186

[nph16050-bib-0044] Niklas KJ . 1985 The aerodynamics of wind pollination. Botanical Review 51: 328–386.

[nph16050-bib-0045] Obeso JR . 2002 The costs of reproduction in plants. New Phytologist 155: 321–348. 10.1046/j.1469-8137.2002.00477.x 33873312

[nph16050-bib-0046] Okubo A , Levin SA . 1989 A theoretical framework for data analysis of wind dispersal of seeds and pollen. Ecology 70: 329–338.

[nph16050-bib-0047] Pickup M , Barrett SCH . 2012 Reversal of height dimorphism promotes pollen and seed dispersal in a wind‐pollinated dioecious plant. Biology Letters 8: 245–248. 22048889 10.1098/rsbl.2011.0950 PMC3297408

[nph16050-bib-0048] Pickup M , Barrett SCH . 2013 The influence of demography and local mating environment on sex ratios in a wind‐pollinated dioecious plant. Ecology and Evolution 3: 629–639. 23532761 10.1002/ece3.465 PMC3605851

[nph16050-bib-0049] Poissant J , Wilson AJ , Coltman DW . 2010 Sex‐specific genetic variance and the evolution of sexual dimorphism: a systematic review of cross‐sex genetic correlations. Evolution 64: 97–107. 19659596 10.1111/j.1558-5646.2009.00793.x

[nph16050-bib-0050] R Core Team . 2018 R: A language and environment for statistical computing. Vienna, Austria: R Foundations for Statistical Computing http://www.R-project.org/.

[nph16050-bib-0051] Rice WR . 1984 Sex chromosomes and the evolution of sexual dimorphism. Evolution 38: 735–742. 28555827 10.1111/j.1558-5646.1984.tb00346.x

[nph16050-bib-0052] Sánchez Vilas J , Pannell JR . 2011 Sexual dimorphism in resource acquisition and deployment: both size and timing matter. Annals of Botany 107: 119–126. 20980325 10.1093/aob/mcq209 PMC3002469

[nph16050-bib-0053] Smith BW . 1963 The mechanism of sex determination in *Rumex hastatulus* . Genetics 48: 1265–1288. 17248184 10.1093/genetics/48.10.1265 PMC1210418

[nph16050-bib-0054] Soons MB , Heil GW , Nathan R , Katul GG . 2004 Determinants of long‐distance seed dispersal by wind in grasslands. Ecology 85: 3056–3068.

[nph16050-bib-0055] Stehlik I , Barrett SCH . 2006 Pollination intensity influences sex ratios in dioecious *Rumex nivalis*, a wind‐pollinated plant. Evolution 60: 1207–1214. 16892971

[nph16050-bib-0056] Stehlik I , Friedman J , Barrett SCH . 2008 Environmental influence on primary sex ratio in a dioecious plant. Proceedings of the National Academy of Sciences, USA 105: 10847–10852. 10.1073/pnas.0801964105 PMC2504832 18658241

[nph16050-bib-0057] Steven JC , Delph LF , Brodie ED . 2007 Sexual dimorphism in the quantitative‐genetic architecture of floral, leaf, and allocation traits in *Silene latifolia* . Evolution 61: 42–57. 17300426 10.1111/j.1558-5646.2007.00004.x

[nph16050-bib-0058] Steven JC , Waller DM . 2007 Isolation affects reproductive success in low‐density but not high‐density populations of two wind‐pollinated *Thalictrum* species. Plant Ecology 190: 131–141.

[nph16050-bib-0059] Tackenberg O , Poschlod P , Bonn S . 2003 Assessment of wind dispersal potential in plant species. Ecological Monographs 73: 191–205.

[nph16050-bib-0060] Teitel Z , Pickup M , Field DL , Barrett SCH . 2016 The dynamics of resource allocation and costs of reproduction in a sexually dimorphic, wind‐pollinated dioecious plant. Plant Biology 18: 98–103. 25865555 10.1111/plb.12336

[nph16050-bib-0061] Thomson FJ , Moles AT , Auld TD , Kingsford RT . 2011 Seed dispersal distance is more strongly correlated with plant height than with seed mass. Journal of Ecology 99: 1299–1307.

[nph16050-bib-0062] Tonnabel J , David P , Klein EK , Pannell JR . 2019 Sex‐specific selection on plant architecture through “budget” and “direct” effects in experimental populations of the wind‐pollinated herb, *Mercurialis annua* . Evolution 73: 897–912. 30852845 10.1111/evo.13714

[nph16050-bib-0063] Weller SG , Sakai AK , Culley TM , Campbell DR , Dunbar‐Wallis AK . 2006 Predicting the pathway to wind pollination: heritabilities and genetic correlations of inflorescence traits associated with wind pollination in *Schiedea salicaria* (Caryophyllaceae). Journal of Evolutionary Biology 19: 331–342. 16599909 10.1111/j.1420-9101.2005.01038.x

