# Supplementary material for: Variation in sexual dimorphism in a wind‐pollinated plant: the influence of geographical context and life‐cycle dynamics
Source: New Phytol. 2019 Aug 12;224(3):1108–20. doi: 10.1111/nph.16050 (PMC6851585; doi:10.1111/nph.16050)
Supplement: Supplementary file 1 — Fig. S1 The geographic distribution of the 30 sampled populations of Rumex hastatulus across the southern USA. Fig. S2 Plant height across life‐cycle stages and populations of Rumex hastatulus. Fig. S3 Sexual dimorphism (%SD) among populations at wk 4 and wk 8 in Rumex hastatulus. Fig. S4 Bioclimatic variables across the geographical range of Rumex hastatulus. Table S1 Summary of univariate results for common glasshouse study of Rumex hastatulus. Table S2 Variation in sexual dimorphism across geographical gradients for populations of Rumex hastatulus. Table S3 Sexual dimorphism and sex‐specific trait mean variation along climatic gradients for populations of Rumex hastatulus. [file NPH-224-1108-s001.pdf]

New Phytologist Supporting Information

Article title: Variation in sexual dimorphism in a wind-pollinated plant: the influence of geographical context and life-cycle dynamics

Authors: Gemma Puixeu, Melinda Pickup, David L. Field, Spencer C. H. Barrett

The following Supporting Information is available for this article:

Figure S1. The geographic distribution of the 30 sampled populations of *Rumex hastatulus* across the southern USA

Figure S2. Plant height across life-cycle stages and populations of *Rumex hastatulus*

Figure S3. Sexual dimorphism (%SD) among populations at (a) week 4 and (b) week 8 in *Rumex hastatulus*

Figure S4. Bioclimatic variables across the geographical range of *Rumex hastatulus*

Table S1. Summary of univariate results for common glasshouse study of *Rumex hastatulus*

Table S2. Variation in sexual dimorphism across geographical gradients for populations of *Rumex hastatulus*

Table S3. Sexual dimorphism and sex-specific trait mean variation along climatic gradients for populations of *Rumex hastatulus*

Figure S1. The geographic distribution of the 30 sampled populations of *Rumex hastatulus* across the southern USA representing the Texas (open circles) and North Carolina (closed circles) chromosome races.

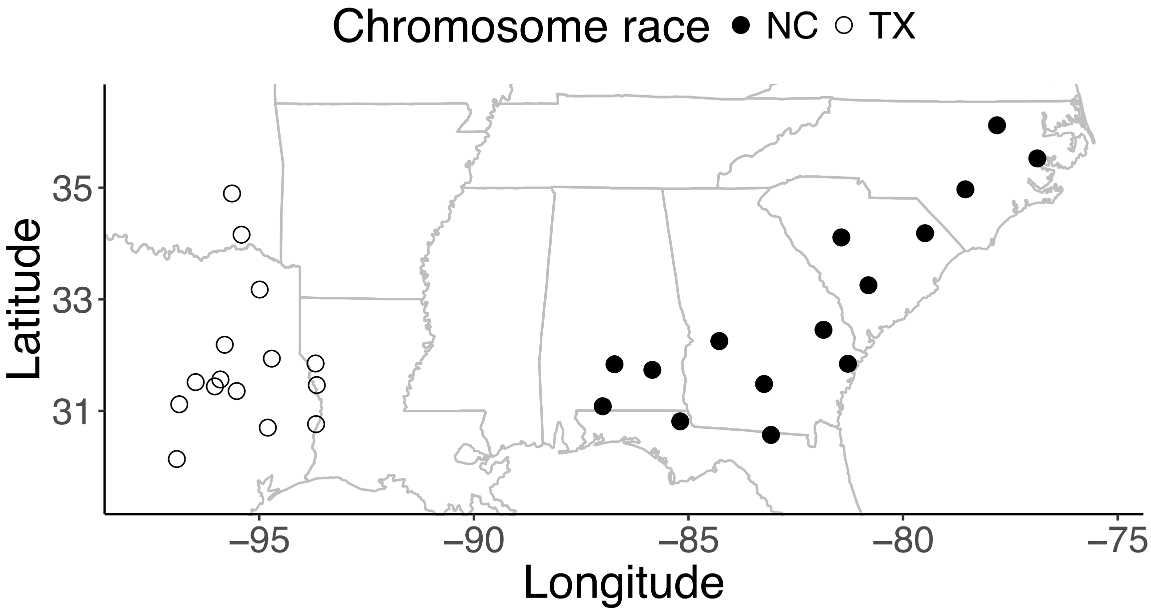

Figure S2. Plant height across life-cycle stages and populations of *Rumex hastatulus*. Predicted means and 95% confidence intervals for males (orange squares) and females (green circles) for each population (individual points) and overall values for each sex (dashed lines and color shading) at weeks 2, 4 and 8. Significance of sex differences per population and overall is indicated with stars above individual bars and at the lower right corner, respectively. * 0.01 < P < 0.05, ** 0.001 < P < 0.01, *** P <0.001. ns (or absence of asterisks on individual points) = not statistically significant.

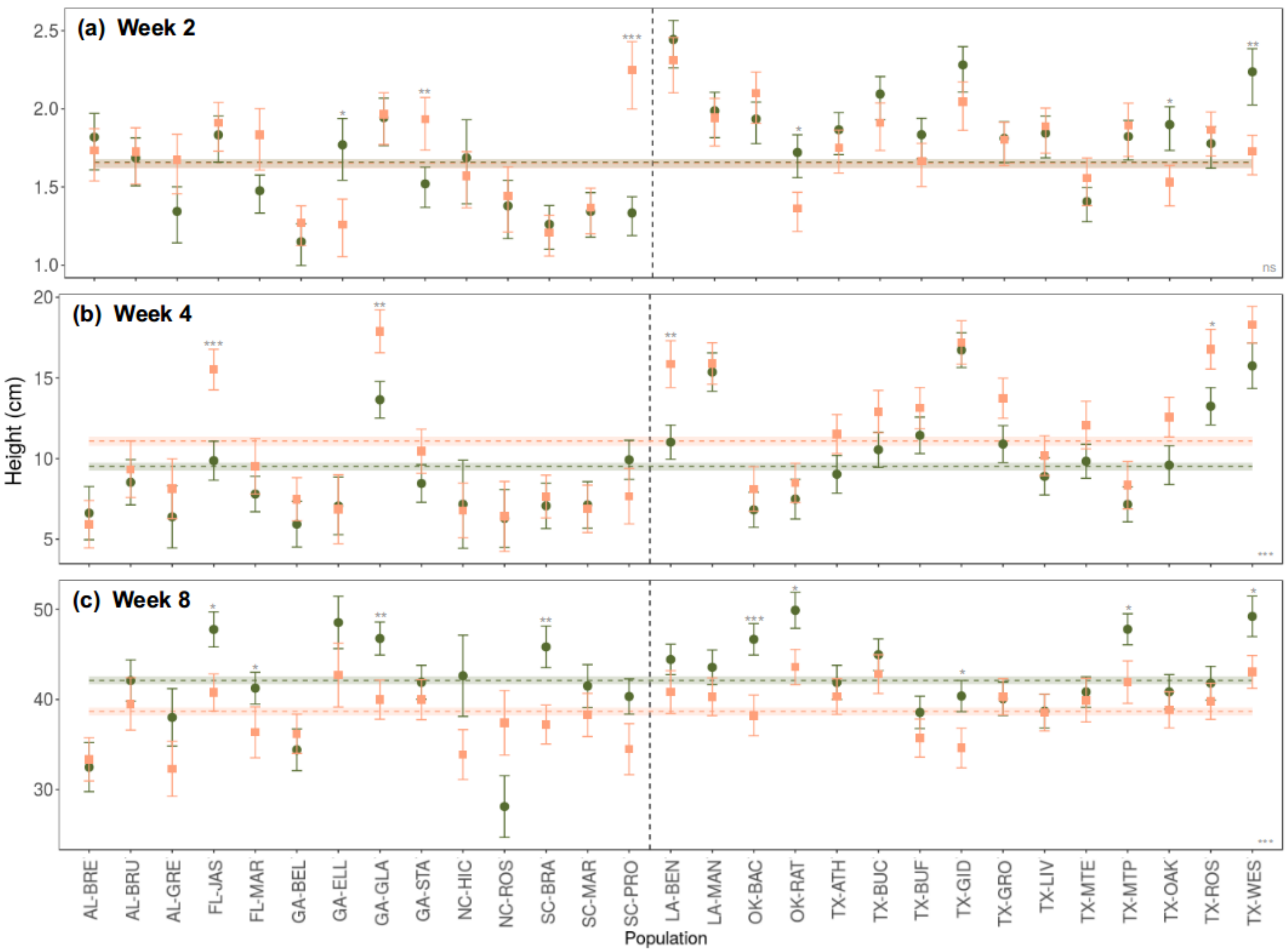

Figure S3. Sexual dimorphism (%SD) among populations at (a) week 4 and (b) week 8 in *Rumex hastatulus*. Percent sexual dimorphism (see the Materials and Methods section) for each population plotted according to its geographical location (longitude and latitude). Green (orange) indicates female (male) bias. Significant variation between sexes and among populations and their interaction obtained from GLMM (see the Materials and Methods section) is indicated as “S”, “P” and “SxP” in the upper left corner, respectively.

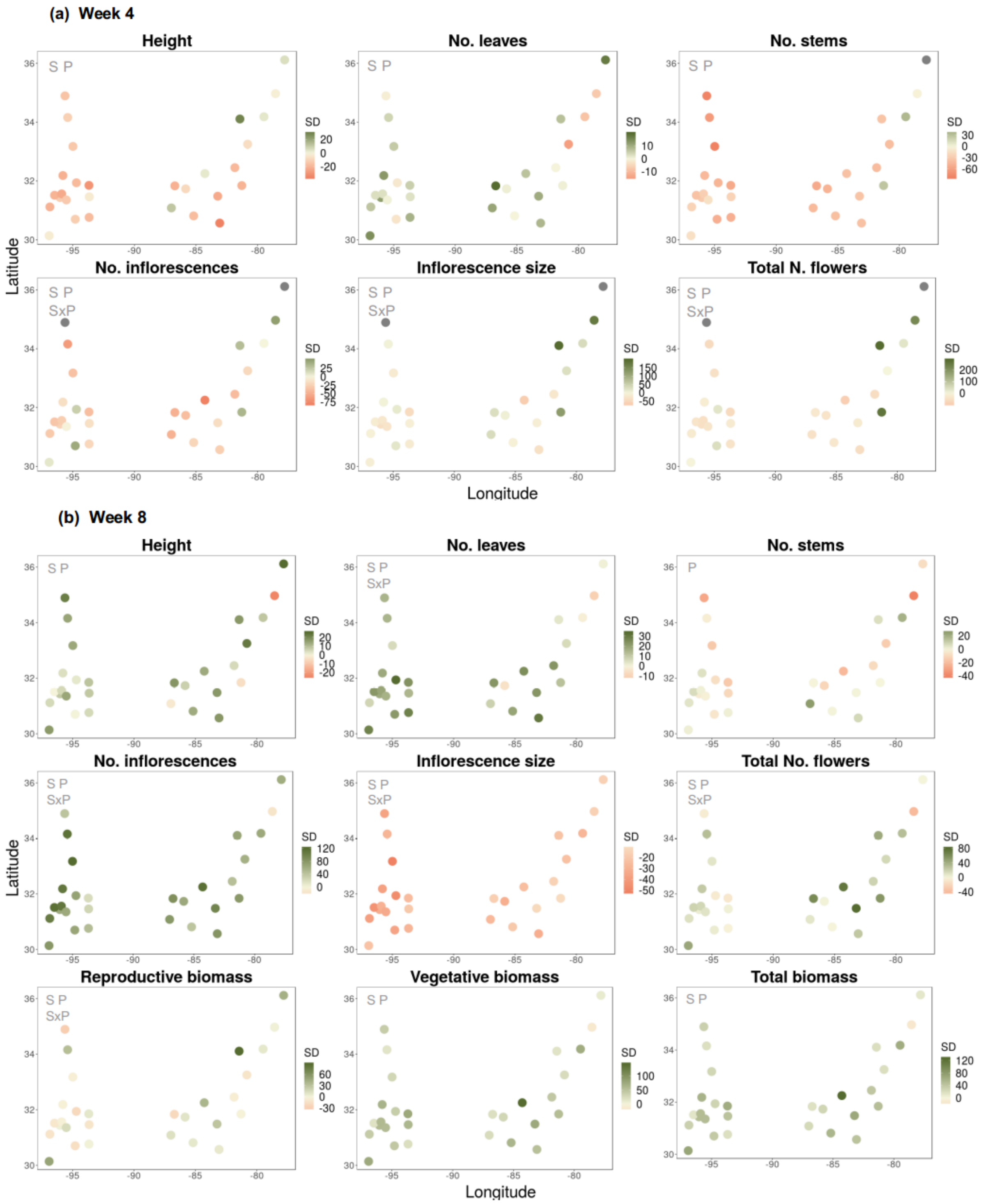

Figure S4. Bioclimatic variables across the geographical range of *Rumex hastatulus*. (a) Matrix showing the patterns of Spearman rank correlations among bioclimatic variables extracted from WorldClim (see the Materials and Methods section). The specifications of each one are as follows; Bio1: annual mean temperature. Bio2: mean diurnal range (mean of monthly (max temp - min temp)). Bio3: isothermality (bio2/bio7)(*100). Bio4: temperature seasonality (standard deviation *100). Bio5: max temperature of warmest month. Bio6: min temperature of coldest month. Bio7: temperature annual range (Bio5-Bio6). Bio8: mean temperature of wettest quarter. Bio9: mean temperature of driest quarter. Bio10: mean temperature of warmest quarter. Bio11: mean temperature of coldest quarter. Bio12: annual precipitation. Bio13: precipitation of wettest month. Bio14: precipitation of driest month. Bio15: precipitation seasonality (coefficient of variation). Bio16: precipitation of wettest quarter. Bio17: precipitation of driest quarter. Bio18: precipitation of warmest quarter. Bio19: precipitation of coldest quarter global potential evapotranspiration (mm). Pet.avg: global potential evapotranspiration (mm). Bio1, Bio7 and Bio12 (green squares) are the variables we used for further analyses. (b) Populations of the North Carolina (green) and Texas (orange) chromosome races projected in the first two principal components from all bioclimatic variables (left) and in the three bioclimatic variables used in the models (right). The contribution of each principal component to the total variance is indicated in the x and y axis respectively. (c) Patterns of Spearman rank correlations between the bioclimatic variables used in the models and geographical parameters. In (a) and (c) color indicates correlation coefficient (blue for positive and red for negative). * $0.01 < P < 0.05$, ** $0.001 < P < 0.01$, *** $P < 0.001$

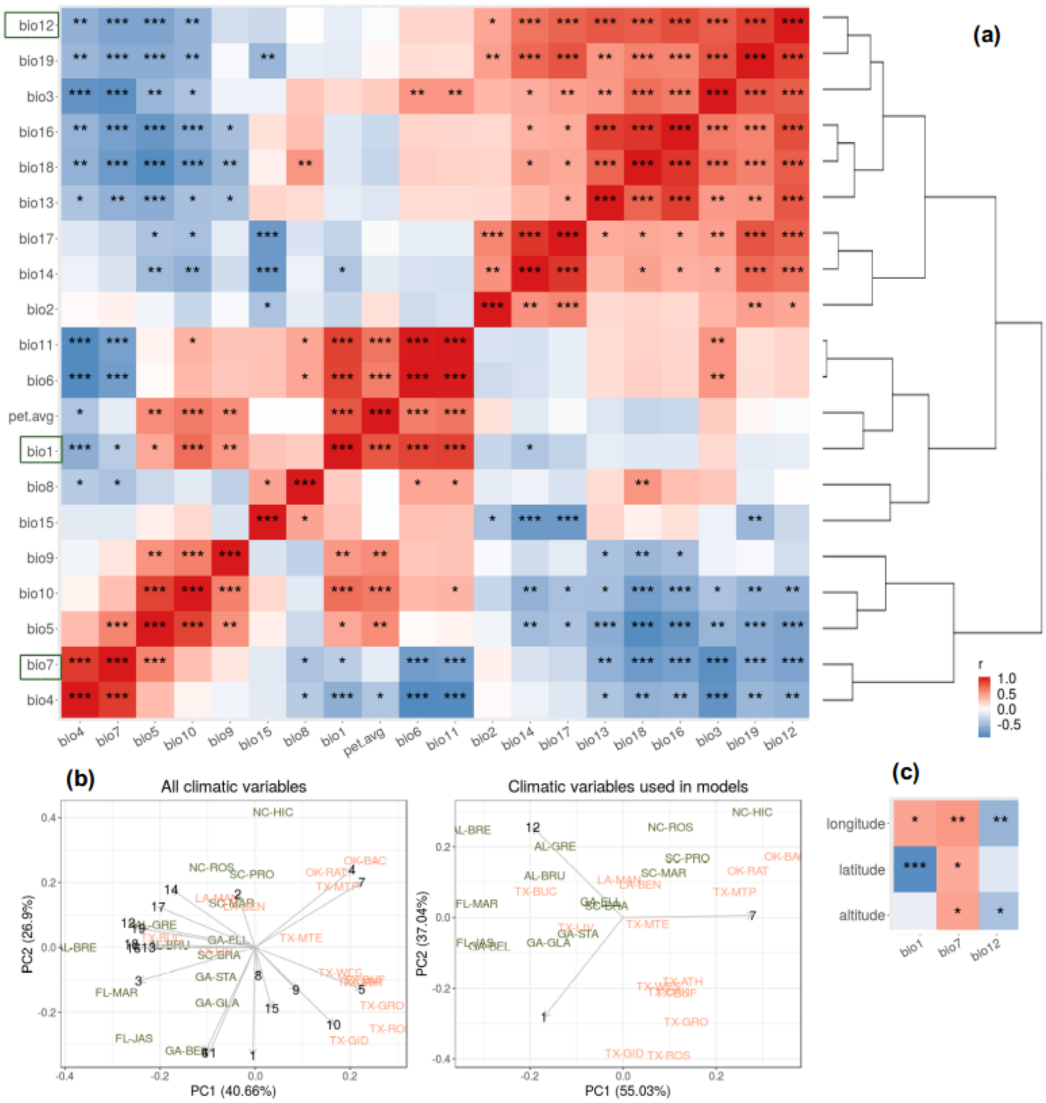

(a)
bio12
bio19
bio3
bio16
bio18
bio13
bio17
bio14
bio2
bio11
bio6
pet.avg
bio1
bio8
bio15
bio9
bio10
bio5
bio7
bio4
r
1.0
0.5
0.0
-0.5
(b)
All climatic variables
Climatic variables used in models
PC1 (40.66%)
PC2 (26.9%)
PC1 (55.03%)
PC2 (37.04%)
(c)
longitude
latitude
altitude
bio1
bio7
bio12

Table S1. Summary of univariate results for common glasshouse study of *Rumex hastatulus*. For each trait, “Data” included in the specific model, “Probability distribution and link function” used for the GLMM that best fitted the data (see the Materials and Methods section). Significance of each “Term” of the model was assessed with ANOVA type 2, which outputted the respective “Chisq” and “P”-value with the specified degrees of freedom (“DF”). The traits considered are height (cm), number of leaves, leaf size (cm), number of stems, flowering (binary: yes or no. Excluded at week 8, since all plants are flowering), proportion of flowering stems (including only flowering individuals), number of inflorescences, inflorescence size (mm, average of 3 inflorescences measured per plant), total flower number (inflorescence number x size; as an integrative flowering measure) and biomass (reproductive, vegetative and total). See the Materials and Methods section for more information. In green, models used in Fig. 1; in blue, models used in Fig. 2; in orange, models used in Fig. S3; highlighted in yellow, significant sex-by-population interactions; in bold, significant results ($P < 0.05$).

Height

Data	Probability distribution and link function	Term	DF	Chisq	P
Overall model	gaussian<br>log link	sex<br>chr.race<br>timepoint<br>sex:chr.race<br>sex:timepoint<br>chr.race:timepoint<br>sex:chr.race:timepoint	1<br>1<br>2<br>1<br>2<br>2<br>2	63.7469309<br>6.5934002<br>6521.559524<br>1.2646441<br>60.9566951<br>26.9118293<br>0.6109309	**1.414738e-15**<br>**1.023575e-02**<br>**0.000000e+00**<br>2.607739e-01<br>**5.799918e-14**<br>**1.432750e-06**<br>7.367804e-01
Week 2<br>sex:chr.race	gaussian<br>log link	sex<br>chr.race<br>sex:chr.race	1<br>1<br>1	1.398911<br>8.883597<br>10.681468	0.236906072<br>**0.002877440**<br>**0.001082142**
Week 2<br>sex in chrNC	gaussian<br>log link	sex	1	5.758235	**0.01641**
Week 2<br>sex in chrTX	gaussian<br>log link	sex	1	7.0128	**0.008093**
Week 2<br>sex:population	gaussian<br>log link	sex<br>population<br>sex:population	1<br>28<br>28	0.9044198<br>120.4373685<br>56.8418676	3.415994e-01<br>**1.962462e-13**<br>**1.014406e-03**
Week 2<br>sex:population<br>in chrNC	gaussian<br>log link	sex<br>population<br>sex:population	1<br>13<br>13	7.756766<br>42.041275<br>31.387678	**0.00535116836**<br>**0.00006448248**<br>**0.00295804878**
Week 2<br>sex:population<br>in chrTX	gaussian<br>log link	sex<br>population<br>sex:population	1<br>14<br>14	6.673605<br>51.331086<br>18.475703	**0.009785106436**<br>**0.000003647968**<br>0.185965556560
Week 4 and 8<br>sex:chr.race	gaussian<br>identity link	sex<br>chr.race<br>timepoint<br>sex:chr.race<br>sex:timepoint<br>chr.race:timepoint<br>sex:chr.race:timepoint	1<br>1<br>1<br>1<br>1<br>1<br>1	6.51473643<br>9.67085386<br>9534.27017079<br>1.18668734<br>75.28170565<br>1.91703140<br>0.08981531	**1.069842e-02**<br>**1.872143e-03**<br>**0.000000e+00**<br>2.759988e-01<br>**4.081237e-18**<br>1.661843e-01<br>7.644121e-01
Week 4<br>sex:chr race	gaussian<br>log link	sex<br>chr.race<br>sex:chr.race	1<br>1<br>1	49.3117208<br>13.0041815<br>0.7050846	**2.183540e-12**<br>**3.107962e-04**<br>4.010805e-01
Week 4<br>sex:population	gaussian<br>identity link	sex<br>population<br>sex:population	1<br>28<br>28	34.01873<br>306.96430<br>25.84712	**5.458412e-09**<br>**1.014322e-48**<br>5.814477e-01
Week 8<br>sex:chr race	gaussian<br>identity link	sex<br>chr.race<br>sex:chr.race	1<br>1<br>1	43.5352902<br>3.4805425<br>0.4136996	**4.163823e-11**<br>6.209438e-02<br>5.200977e-01
Week 8<br>sex:population	gaussian<br>identity link	sex<br>population<br>sex:population	1<br>28<br>28	42.84307<br>124.38003<br>34.31265	**5.931183e-11**<br>**4.120350e-14**<br>1.907682e-01

Number of leaves

Data	Probability distribution and link function	Term	DF	Chisq	P

Overall model	poisson<br>log link	sex<br>chr.race<br>timepoint<br>sex:chr.race<br>sex:timepoint<br>chr.race:timepoint<br>sex:chr.race:timepoint	1<br>1<br>2<br>1<br>2<br>2<br>2	73.416745<br>7.270067<br>22192.361799<br>2.974602<br>75.198294<br>731.792670<br>7.597839	**1.049718e-17**<br>**7.011317e-03**<br>**0.000000e+00**<br>8.458094e-02<br>**4.687032e-17**<br>**1.239484e-159**<br>**2.239496e-02**
Week 2<br>sex:chr.race	poisson<br>log link	sex<br>chr.race<br>sex:chr.race	1<br>1<br>1	1.7288694<br>119.2117480<br>0.1774768	1.885554e-01<br>**9.412581e-28**<br>6.735506e-01
Week 2<br>sex:population	poisson<br>log link	sex<br>population<br>sex:population	1<br>28<br>28	1.2058196<br>187.9635<br>10.5318687	0.2721616<br>**1.268683e-25**<br>0.9988671
Week 4 and 8<br>sex:chr.race	poisson<br>log link	sex<br>chr.race<br>timepoint<br>sex:chr.race<br>sex:timepoint<br>chr.race:timepoint<br>sex:chr.race:timepoint	1<br>1<br>1<br>1<br>1<br>1<br>1	89.02813<br>1.3142692<br>654.9329<br>8.618317291<br>30.176146505<br>233.9688<br>1.240379	**3.892376e-21**<br>0.2516231<br>**1.889297e-144**<br>**0.003327991**<br>**0.0000000394532**<br>**8.125592e-53**<br>0.265398
Week 4<br>sex:chr.race	poisson<br>log link	sex<br>chr.race<br>sex:chr.race	1<br>1<br>1	12.6242005920<br>84.43197<br>0.7229596	**0.0003807847**<br>**3.976658e-20**<br>0.3951748
Week 4<br>sex:population	poisson<br>log link	sex<br>population<br>sex:population	1<br>28<br>28	12.7083088276<br>670.6441 30.1928560	**0.0003640345**<br>**2.662750e-123**<br>0.3540585
Week 8<br>sex:chr.race	poisson<br>log link	sex<br>chr.race<br>sex:chr.race	1<br>1<br>1	207.2982e<br>2.3678743<br>6.954425910	**5.337548e-47**<br>0.1238556<br>**0.008361212**
Week 8<br>sex:chr.race<br>in chrNC	poisson<br>log link	sex	1	44.646	**2.361e-11**
Week 8<br>sex:chr.race<br>in chrTX	poisson<br>log link	sex	1	170.09	**7.071646e-39**
Week 8<br>sex:population	poisson<br>log link	sex<br>population<br>sex:population	1<br>28<br>28	205.6912<br>313.1892<br>66.214854104	**1.196685e-46**<br>**5.848013e-50**<br>**0.00006179246**
Week 8<br>sex:population<br>in chrNC	poisson<br>log link	sex<br>population<br>sex:population	1<br>13<br>13	44.89326<br>68.1016174969<br>38.9824332442	**2.080726e-11**<br>**0.0000000017914**<br>**0.0002012385**
Week 8<br>sex:population<br>in chrTX	poisson<br>log link	sex<br>population<br>sex:population	1<br>14<br>14	168.6908<br>240.0367<br>20.2902589	**1.429244e-38**<br>**3.287855e-43**<br>0.1212526

Leaf size

Data	Probability distribution and link function	Term	DF	Chisq	P
Overall model (weeks 4 and 8)	gaussian<br>identity link	sex<br>chr.race<br>timepoint	1<br>1<br>1	21.179459869559<br>0.3881972<br>18.89039239322	**0.000004182227**<br>0.5332486<br>**0.00001384482**

		sex:chr.race<br>sex:timepoint<br>chr.race:timepoint<br>sex:chr.race:timepoint	1<br>1<br>1<br>1	0.4726430<br>16.53367156729<br>174.192938878060<br>0.02318004	0.4917738<br>**0.00004779377**<br>**8.983599e-20**<br>0.87898982
Week 4<br>sex:chr.race	gamma<br>log link	sex<br>chr.race<br>sex:chr.race	1<br>1<br>1	1.9209890<br>14.8741446736<br>3.21651121	0.1657477<br>**0.0001149275**<br>0.07289885
Week 4<br>sex:population	gamma<br>log link	sex<br>population<br>sex:population	1<br>26<br>26	1.8308243<br>134.6411<br>31.5174769	0.1760302<br>**6.699526e-16**<br>0.2945879
Week 8<br>sex:chr.race	gamma<br>log link	sex<br>chr.race<br>sex:chr.race	1<br>1<br>1	30.15011275927874<br>4.61881143<br>0.1594230	**0.00000003998639**<br>**0.03162314**<br>0.6896883
Week 8<br>sex:population	gamma<br>log link	sex<br>population<br>sex:population	1<br>28<br>28	30.27703310106227<br>110.5428<br>25.8315415	**0.00000003745346**<br>**9.281919e-12**<br>0.5823039

Number of stems

Data	Probability distribution and link function	Term	DF	Chisq	P
Overall model (weeks 4 and 8)	poisson<br>log link	sex<br>chr.race<br>timepoint<br>sex:chr.race<br>sex:timepoint<br>chr.race:timepoint<br>sex:chr.race:timepoint	1<br>1<br>1<br>1<br>1<br>1<br>1	25.06500340<br>34.574535198<br>1614.186 0.2770829<br>47.44892<br>138.8962 0.6449624	**0.0000005542978**<br>**0.0000000041023**<br>**0.000**<br>0.5986196<br>**5.645595e-12**<br>**4.640930e-32**<br>0.4219195
Week 4<br>sex:chr.race	poisson<br>log link	sex<br>chr.race<br>sex:chr.race	1<br>1<br>1	70.47907<br>24.792432923<br>658 0.2550384	**4.651755e-17**<br>**0.0000006384763**<br>0.6135495
Week 4<br>sex:population	poisson<br>log link	sex<br>population<br>sex:population	1<br>27<br>27	63.90558<br>335.3494<br>29.3300887	**1.305272e-15**<br>**6.108677e-55**<br>0.3450777
Week 8<br>sex:chr.race	poisson<br>log link	sex<br>chr.race<br>sex:chr.race	1<br>1<br>1	1.169665<br>34.902336126<br>0.2554038	0.279470<br>**0.000000003466**60.6132955
Week 8<br>sex:population	poisson<br>log link	sex<br>population<br>sex:population	1<br>28<br>28	1.5619531<br>123.0334<br>18.1040272	0.2113795<br>**7.032193e-14**<br>0.9234987

Flowering

Data	Probability distribution and link function	Term	DF	Chisq	P
Overall model (weeks 4 and 8)	binomial<br>logit link	sex<br>chr.race<br>timepoint<br>sex:chr.race<br>sex:timepoint<br>chr.race:timepoint<br>sex:chr.race:timepoint	1<br>1<br>1<br>1<br>1<br>1<br>1	218875.1 24046.87<br>>1e+10<br>5140.366 >1e+10<br>>1e+10<br>>1e+10	**1e<-16**<br>**1e<-16**<br>**1e<-16**<br>**1e<-16**<br>**1e<-16**<br>**1e<-16**<br>**1e<-16**

Week 4<br>sex:chr.race	gaussian<br>identity link	sex<br>chr.race<br>sex:chr.race	1<br>1<br>1	1.229424e+02<br>1.3760826 0.7082089	**1.435582e-28**<br>0.2407696<br>0.4000391
Week 4<br>sex:population	gaussian<br>identity link	sex<br>population<br>sex:population	1<br>27<br>27	1.234959e+02<br>79.372986333675<br>33.1143852	**1.086132e-28**<br>**0.000000469827**<br>0.1933213

Proportion of flowering stems

Data	Probability distribution and link function	Term	DF	Chisq	P
Overall model (weeks 4 and 8)	binomial<br>logit link	sex<br>chr.race<br>timepoint<br>sex:chr.race<br>sex:timepoint<br>chr.race:timepoint<br>sex:chr.race:timepoint	1<br>1<br>1<br>1<br>1<br>1<br>1	20.498521424223<br>0.02115992<br>8.319732e+01<br>5.98982707<br>0.05774818<br>3.34391227<br>2.1932536	**0.000005967733**<br>0.88434416<br>**7.425829e-20**<br>**0.01438861**<br>0.81009109<br>**0.06745405**<br>0.1386162
Week 4<br>sex:chr.race	gaussian<br>log link	sex<br>chr.race<br>sex:chr.race	1<br>1<br>1	7.41244915556<br>2.1013490<br>7.455491328	**0.00003008491**<br>0.1471692<br>**0.006324321**
Week 4<br>sex in chrNC	gaussian<br>log link	sex	1	0.1566383	0.6922706
Week 4<br>sex in chrTX	gaussian<br>log link	sex	1	23.333985962420	**0.000001361725**
Week 4<br>sex:population	gaussian<br>identity link	sex<br>population<br>sex:population	1<br>27<br>27	6.390558e+01<br>3.353494e+02<br>29.3300887	**1.305272e-15**<br>**6.108677e-55**<br>0.3450777
Week 4<br>sex:population<br>chrNC	gaussian<br>identity link	sex<br>population<br>sex:population	1<br>4<br>4	0.01251367<br>3.4241860 3.6814761	0.91093084<br>0.4894992<br>0.4508259
Week 4<br>sex:population<br>chrTX	gaussian<br>identity link	sex<br>population<br>sex:population	1<br>11<br>11	27.7445413269345<br>7.8970446<br>13.2382196	**0.000000138438**<br>0.7224957<br>0.2780411
Week 8<br>sex:chr.race	gaussian<br>identity link	sex<br>chr.race<br>sex:chr.race	1<br>1<br>1	9.123334479<br>0.01248944<br>0.3001888	**0.002523695**<br>0.91101675<br>0.5837641
Week 8<br>sex:population	gaussian<br>identity link	sex<br>population<br>sex:population	1<br>28<br>28	8.390999467<br>28.3408332<br>50.28895921	**0.003770835**<br>0.4465005<br>**0.00600363**

Number of inflorescences (of already flowering individuals)

Data	Probability distribution and link function	Term	DF	Chisq	P
Overall model (weeks 4 and 8)	posison<br>log link	sex<br>chr.race<br>timepoint<br>sex:chr.race<br>sex:timepoint<br>chr.race:timepoint	1<br>1<br>1<br>1<br>1<br>1	4.423842e+02<br>12.4220357541<br>4368.343<br>1.1690967<br>2.281527e+02<br>5.814865e+01	**3.277623e-98**<br>**0.0004242975**<br>**0.000**<br>0.2795869<br>**1.507389e-51**<br>**2.430404e-14**

		sex:chr.race:timepoint	1	0.5451541	0.4603045
Week 4<br>sex:chr.race	poisson<br>log link	sex<br>chr.race<br>sex:chr.race	1<br>1<br>1	18.07224375778<br>3.03020004<br>0.2635288	**0.00002126793**<br>0.08172794<br>0.6077061
Week 4<br>sex:population	poisson<br>log link	sex<br>population<br>sex:population	1<br>26<br>26	19.17633499543<br>51.350271779<br>33.282751	**0.00001191818**<br>**0.002161479**<br>0.154052
Week 8<br>sex:chr.race	poisson<br>log link	sex<br>chr.race<br>sex:chr.race	1<br>1<br>1	1497.218<br>12.2044047406<br>3.060074	**0.000**<br>**0.0004767681**<br>0.080238
Week 8<br>sex:population	poisson<br>log link	sex<br>population<br>sex:population	1<br>28<br>28	1.474375e+03<br>1.398100e+02<br>1.600115e+02	**1.432790e-322**<br>**8.178347e-17**<br>**1.888116e-20**

Inflorescence size

Data	Probability distribution and link function	Term	DF	Chisq	P
Overall model (weeks 4 and 8)	gaussian<br>log link	sex<br>chr.race<br>timepoint<br>sex:chr.race<br>sex:timepoint<br>chr.race:timepoint<br>sex:chr.race:timepoint	1<br>1<br>1<br>1<br>1<br>1<br>1	147.0124<br>13.6371083489<br>4826.634 8.30738953<br>12.4552880108<br>33.308022658002180<br>0.1530384	**7.798701e-34**<br>**0.0002217587**<br>**1e<-16**<br>**0.00394841 0.0004168101**<br>**0.000000007865747**<br>**0.6956485**
Week 4<br>sex:chr.race	gaussiain<br>log link	sex<br>chr.race<br>sex:chr.race	1<br>1<br>1	17.47593071722<br>25.2750771779522<br>3.9829281	**0.00002909682**<br>**0.0000004970905**<br>**0.0459636**
Week 4<br>sex in chrNC	gaussian<br>log link	sex	1	0.2454831	0.6202737
Week 4<br>sex in chrTX	gaussian<br>log link	sex	1	18.45961013566	**0.00001735435**
Week 4<br>sex:population	gamma<br>log link	sex<br>population<br>sex:population	1<br>26<br>26	13.702738148<br>78.4749562801994<br>45.61025616	**0.000214142**<br>**0.0000003594530.01008064**
Week 4<br>sex:population<br>chrNC	gamma<br>log link	sex<br>population<br>sex:population	1<br>1<br>1	0.2017679<br>29.244383789<br>34.0904502028	0.6532976 **0.003623662**<br>**0.0006527382**
Week 4<br>sex:population<br>chrTX	gamma<br>log link	sex<br>chr.race<br>sex:chr.race	1<br>1<br>1	0.2017679<br>29.244383789<br>34.0904502028	**0.6532976 0.003623662**<br>**0.0006527382**
Week 8<br>sex:chr.race	gamma<br>log link			3.246676e+02<br>13.0536850885<br>32.00790194916922	**1.393839e-72**<br>**0.0003026883**<br>**0.000000015354**
Week 8<br>sex chrNC	gamma<br>log link	sex	1	4.203878e+01	**8.948107e-11**
Week 8<br>sex chrTX	gamma<br>log link	sex	1	2.915468e+02	**2.288311e-65**

Week 8<br>sex:population	gamma<br>log link	sex<br>population<br>sex:population	1<br>28<br>28	308.2576<br>216.2465<br>48.973760740	**5.233551e-69**<br>**5.550972e-31**<br>**0.008399235**
Week 8<br>sex:population<br>chrNC	gamma<br>log link	sex<br>population<br>sex:population	1<br>13<br>13	44.57819<br>43.166615154<br>6.0426625	**2.443994e-11**<br>**0.00004214201**<br>0.9445865
Week 8<br>sex:population<br>chrTX	gamma<br>log link	sex<br>population<br>sex:population	1<br>14<br>14	301.6316<br>121.5143<br>27.7636863	**1.453149e-67**<br>**3.177501e-19**<br>**0.0152908**

Estimated total flower number

Data	Probability distribution and link function	Term	DF	Chisq	P
Overall model (weeks 4 and 8)	gaussian<br>log link	sex<br>chr.race<br>timepoint<br>sex:chr.race<br>sex:timepoint<br>chr.race:timepoint<br>sex:chr.race:timepoint	1<br>1<br>1<br>1<br>1<br>1<br>1	0.00735907<br>0.0002322504<br>133.28<br>0.0005282701<br>154.36<br>125.81<br>1.6882950	0.93163731<br>0.9878408974<br>**1e<-16**<br>0.9816629335<br>**1e<-16**<br>**1e<-16**<br>**0.1938259**
Week 4<br>sex:chr.race	gamma<br>log link	sex<br>chr.race<br>sex:chr.race	1<br>1<br>1	23.147304667792<br>11.0180742999<br>0.1352528	**0.000001500534**<br>**0.0009022776**<br>0.7130468
Week 4<br>sex:population	gamma<br>log link	sex<br>population<br>sex:population	1<br>26<br>26	84396.1<br>2985257<br>2247414	**1e<-16**<br>**1e<-16**<br>**1e<-16**
Week 8<br>sex:chr.race	gaussian<br>log link	sex<br>chr.race<br>sex:chr.race	1<br>1<br>1	88.54569<br>1.2545504<br>12.2610210007	**4.967414e-21**<br>0.2626852<br>**0.0004625184**
Week 8<br>sex chrNC	gaussian<br>log link	sex	1	54.109	**1.896e-13**
Week 8<br>sex chrTX	gaussian<br>log link	sex	1	12.081	**0.0005094**
Week 8<br>sex:population	gaussian<br>log link	sex<br>population<br>sex:population	1<br>28<br>28	86.32863<br>136.8871<br>96.565107947120438	**1.523864e-20**<br>**2.692196e-16**<br>**0.000000001812**
Week 8<br>sex:population<br>chrNC	gaussian<br>log link	sex<br>population<br>sex:population	1<br>13<br>13	47.57167<br>54.5243803292198<br>34.8779429371	**5.302924e-12**<br>**0.000000489637**<br>**0.0008840001**
Week 8<br>sex:population<br>chrTX	gaussian<br>log link	sex<br>population<br>sex:population	1<br>14<br>14	12.6518515139<br>103.6572<br>25.6856266	**0.0003751939**<br>**9.407107e-16**<br>**0.0283721**

Biomass (reprod figure + text; veg figure)

Data	Probability distribution and link function	Term	DF	Chisq	P
Reproductive biomass<br>sex:chr.race	gaussian<br>log link	sex<br>chr.race	1<br>1	1.6597645<br>2.96716602	0.1976351<br>0.08497059

		sex:chr.race	1	4.59407313	**0.03208269**
Reproductive biomass<br>sex:chr.race<br>chrNC	gaussian<br>log link	sex	1	4.8975	**0.0269**
Reproductive biomass<br>sex:chr.race<br>chrTX	gaussian<br>log link	sex	1	0.0358	0.8499
Reproductive biomass<br>sex:population	gaussian<br>log link	sex<br>population<br>sex:population	1<br>28<br>28	1.3818597<br>59.1968599805<br>37.8861887	0.2397846<br>**0.0005152761**<br>0.1005714
Reproductive biomass<br>sex:population<br>chrNC	gaussian<br>log link	sex<br>population<br>sex:population	1<br>13<br>13	5.0284297 9.0509717<br>12.9372494	**0.0249345**<br>0.7690831<br>0.4526703
Reproductive biomass<br>sex:population<br>chrTX	gaussian<br>log link	sex<br>population<br>sex:population	1<br>14<br>14	0.1694783<br>44.424483782<br>4 25.06949530	0.6805759<br>**0.00005055958**<br>**0.03388748**
Vegetative biomass<br>sex:chr.race	gamma<br>log link	sex<br>chr.race<br>sex:chr.race	1<br>1<br>1	174.3556<br>1.3031933<br>0.3122547	**8.278210e-40**<br>0.2536308<br>0.5762999
Vegetative biomass<br>sex:population	gamma<br>log link	sex<br>population<br>sex:population	1<br>28<br>28	190.1270<br>127.3601<br>37.2637506	**2.982722e-43**<br>**1.255878e-14**<br>0.1131571
Total biomass<br>sex:chr.race	gamma<br>log link	sex<br>chr.race<br>sex:chr.race	1<br>1<br>1	163.5808<br>0.9036169<br>0.05196842	**1.867832e-37**<br>0.3418137<br>0.81967281
Total biomass<br>sex: population	gamma<br>log link	sex<br>population<br>sex:population	1<br>28<br>28	178.6623<br>122.2484<br>37.2204009	**9.494910e-41**<br>**9.596837e-14**<br>0.1140791

Table S2. Variation in sexual dimorphism across geographical gradients for populations of *Rumex hastatulus*. Results of multiple regression of percent sexual dimorphism (%SD) of different reproductive and vegetative traits at weeks 4 and 8 among populations on altitude, latitude and longitude. Only significant contributions are displayed. Specific models are: No. leaves (week 8) = 160.255 - 4.496 Latitude (R^2 = 0.35, P = 0.0004); No. stems (week 4) = -12.146 - 0.245 Altitude (R^2 = 0.22, P = 0.007); No. stems (week 8) = 117.864 - 3.614 Latitude (R^2 = 0.16, P = 0.0374); No. inflorescences (week 8) = 48.208 + 0.2535 Altitude (R^2 = 0.14, P = 0.027); Inflorescence size (week 4) = -894.198 + 28.31 Latitude (R^2 = 0.29, P = 0.0023); Inflorescence size (week 4) = 441.094 -4.806 Longitude (R^2 = 0.23, P = 0.0065); Inflorescence size (week 8) = 60.5 -0.996 Longitude (R^2 = 0.4261, P = 7.43e-05); Total flower number (week 4) = 602.548 - 6.748 Longitude (R^2 = 0.17, P = 0.018); Total flower number (week 4) = -1121.55 + 35.03 Latitude (R^2 = 0.16, P = 0.022); Total flower number (week 8) = 580.979 + 0.338 Altitude - 10.03 Latitude - 3.044 Longitude (R^2 = 0.33, P = 0.004). Color indicates the direction of the correlation (red for positive and blue for negative). * 0.01 < P < 0.05, ** 0.001 < P < 0.01, *** P <0.001.

Trait	Altitude	Latitude	Longitude
No. leaves (week 8)		***	
No. stems (week 4)	**		
No. stems (week 8)		*	
No. inflorescences (week 8)	*		
Inflorescence size (week 4)		**	**
Inflorescence size (week 8)			**
Total No. flowers (week 4)		*	*
Total No. flowers (week 8)	**	**	**

Table S3. Sexual dimorphism and sex-specific trait mean variation along climatic gradients for populations of *Rumex hastatulus*. For each trait, grey (first) line shows the correlation coefficients and P-values of the significant results of the multiple regression analyses (Table S2): percent sexual dimorphism (%SD) across populations regressed on mean annual temperature, annual temperature range and total annual precipitation (see Fig. S4 for more information on climatic variables). See the Materials and Methods section for more details and Fig. 5 for full models. Green (second) and orange (third) lines show coefficients and *P*-values of regression of respectively female and male trait means on bioclimatic variables.

	Temperature annual mean		Temperature annual range		Precipitation annual	
	Coeff	P	Coeff	P	Coeff	P
Height (week 4)	-0.6145	0.00428				
	0.1312	0.00611				
	0.21363	0.000329				
No. stems (week 4)	-1.11871	0.049	-1.27514	0.000427	-0.12069	0.002103
	0.03362	0.00118	0.00305	0.640	-0.002054	0.00769
	0.04132	0.0039	0.009822	0.261	-0.002409	0.0234
Inflorescence size (week 4)	-3.476	0.00487				
	0.3259	0.00321				
	0.4963	0.00122				
Overall flowering (week 4)	-8.3239	0.000803	-4.0849	0.003361	-0.3566	0.015412
	1.718	0.0124	-0.282	0.496	-0.08364	0.1023
	2.738	0.0199	-0.2174	0.758	-0.10388	0.2379
No. leaves (week 8)	0.5601	0.001084				
	-0.17956	0.047942				
	-0.31876	0.000166				
No. stems (week 8)	0.5468	0.01124				
	0.033839	0.000694				
	0.01145	0.329				
Inflorescence size (week 8)	-0.45049	0.004442	-0.38975	7.19e-05		
	0.07978	0.00603	-0.01445	0.4145		
	0.12474	0.00262	0.01830	0.476		
